# Earliest evidence for invasive mitigation of dental caries by Neanderthals

**DOI:** 10.1371/journal.pone.0347662

**Published:** 2026-05-13

**Authors:** Alisa V. Zubova, Lydia V. Zotkina, John W. Olsen, Alexander M. Kulkov, Vyacheslav G. Moiseyev, Anna A. Malyutina, Roman V. Davydov, Sergey V. Markin, Eugene A. Maksimovskiy, Pavel V. Chistyakov, Andrey I. Krivoshapkin, Ksenia A. Kolobova

**Affiliations:** 1 Department of Anthropology, Peter the Great Museum of Anthropology and Ethnography (Kunstkamera), Russian Academy of Sciences, St. Petersburg, Russia; 2 Institute of Archaeology and Ethnography, Siberian Branch, Russian Academy of Sciences, Novosibirsk, Russia; 3 School of Anthropology, University of Arizona, Tucson, Arizona, United States of America; 4 Research Centre for X-ray Diffraction Studies, Saint Petersburg State University, St. Petersburg, Russia; 5 Institute for the History of Material Culture, Russian Academy of Sciences, St. Petersburg, Russia; 6 Nikolaev Institute of Inorganic Chemistry, Siberian Branch, Russian Academy of Sciences, Novosibirsk, Russia; 7 Archeology and Paleoecology of Stone Age in Central Asia (APSACA), Uzbekistan Academy of Sciences, Tashkent, Uzbekistan; Universita degli Studi di Ferrara, ITALY

## Abstract

Neanderthal medical knowledge has long attracted scholarly interest. Evidence suggests they cared for sick, injured, and elderly group members, with possible use of medicinal plants. However, it remains uncertain whether such practices reflect deliberate medical strategies or instinctive self-medication akin to that observed in non-human primates. Here, we analyze and interpret traces of deliberate artificial manipulation of Chagyrskaya 64, a Neanderthal lower left second molar found in Chagyrskaya Cave (Altai Krai, Russia). The tooth exhibits a large human-generated concavity on the occlusal surface, created during the lifetime of the individual. Traceological and microtomographic analyses of the observed modifications, combined with experimental verification, reveal that the concavity in Chagyrskaya 64 is indicative of the earliest documented instance of caries treatment involving the drilling/rotating with a lithic perforator, ca. 59 ka. Evidence of two distinct types of manipulations requiring different tools, in addition to the drilling/rotating technique, necessitating complex finger movements, indicates that the Chagyrskaya Cave Neanderthals possessed the cognitive capacity to intuit the source of pain, comprehend the feasibility of its elimination, and deliberately select the most efficacious dental intervention. These patterns bring Neanderthal behavior closer to modern humans and differentiate that behavior from the instinctive actions of other primates.

## Introduction

The extent of medical knowledge of Neanderthals has been the subject of close attention from researchers for many years. This study provides insight into both the personal history of individuals and the cognitive abilities of the Neanderthal species. Extant data demonstrate with a high degree of certainty that Neanderthals possessed a deep understanding of their natural environment and its resources, ensuring adaptive flexibility in their dietary habits [1,2] and the possibility of carrying out medical practices related to both self-medication and the care of sick and physically challenged members of their social group [3,4].

A key question in the study of the medical capabilities and practices of *H. neanderthalensis* is whether these behaviors were instinctive, similar to those exhibited by some other higher primates who have been observed using medicinal plants for self-treatment of diseases and parasitic infestations [5], or if they were an advanced adaptive strategy involving the conscious choice of treatment, similar to that of modern humans. The current state of knowledge does not permit the formulation of a definitive conclusion. Although most research indicates the recovery of Neanderthals from injury or disease [3,6–11], it remains impossible to ascertain whether any specific manipulations or interventions were performed.

**Fig 1:**
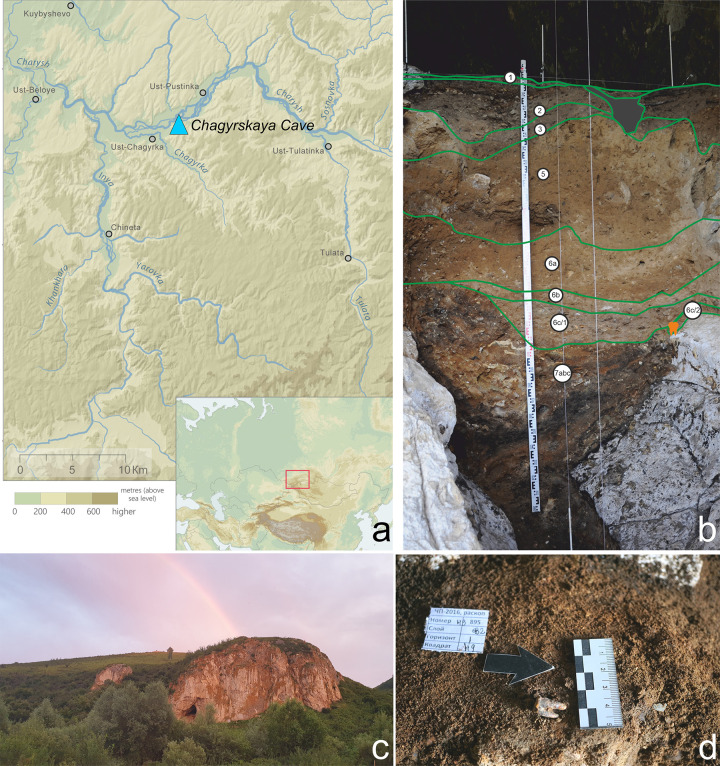
Chagyrskaya Cave, southwestern Siberia, Russia. a. cave location map (created in ArcGIS software, using open data from https://www.usgs.gov/products/maps accessed on December 15, 2021); b. stratigraphic sequence with Chagyrskaya 64 molar discovery location indicated in orange; c. general view of the cave; d. discovery location of the Chagyrskaya 64 molar *in situ* in Layer 6c/2.

**Fig 2:**
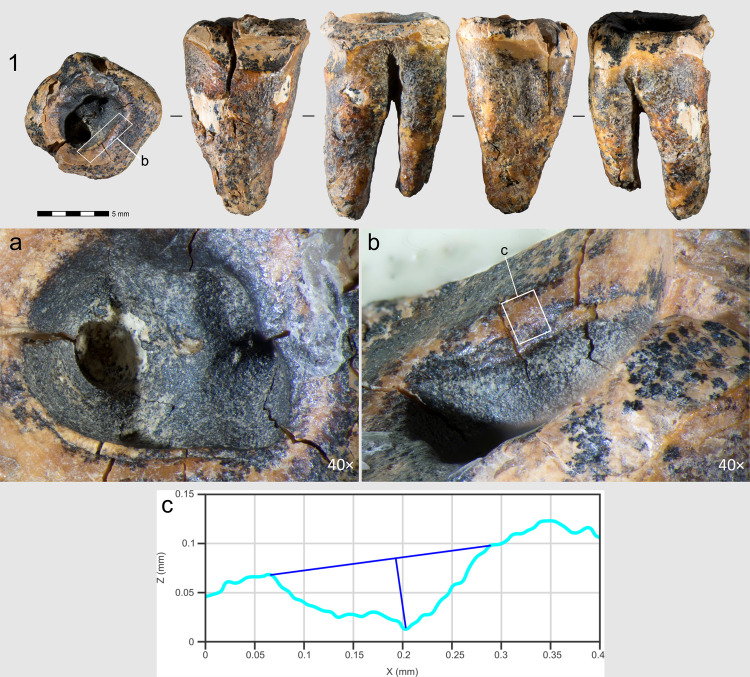
Chagyrskaya 64 molar tooth and its macro-features. 1 General view of the tooth in five projections; a–c. Macro-photographs of the crown’s occlusal surface features: a. superior view of the concavity; b. stepped groove on the concavity’s wall; c. cross-sectional profile of the groove.

The earliest known dental manipulations associated with caries treatment have been described in Upper Paleolithic *Homo sapiens* [12]. The only “medical” actions that have so far been reliably recorded in Neanderthal fossils are the use of toothpicks, presumably aimed not only at removing food particles from the interdental space but also at alleviating the pain of periodontal inflammation [13]. However, traces of toothpick use have been observed not only in Neanderthals, but also in the earliest known member of the genus *Homo*, *H. habilis*, who is thought to have used improvised materials to remove food residues [14], and observed in the Japanese macaque (*Macaca fuscata*) [15]. Therefore, it cannot be concluded that toothpick traces are in themselves a marker of advanced cognitive patterns. Here we provide the results of comprehensive analysis of an unusual concavity on the occlusal surface of a Neanderthal lower molar from Chagyrskaya Сave (Figs 1 and 2), which may represent a case of dental intervention more sophisticated and more invasive than toothpick use.

Chagyrskaya Сave is located in the northwestern Altai Mountains in southwestern Siberia (Fig 1: a, c). The site was occupied by the easternmost of the currently known late Neanderthal populations, which appeared in this area due to in-migration from Central and Eastern Europe around 70−60 ka, and where they persisted at least until 49 ka [16,17]. A total of more than 70 hominin fossils, including 26 dental specimens, have been found during excavations in Chagyrskaya Сave.

The case under discussion here was identified on the lower molar of an adult Neanderthal (designated Chagyrskaya 64). It is a large, irregularly shaped concavity extending to the floor of the pulp chamber (Fig 2: 1, a). The main goal of our study was to establish whether the formation of this concavity was artificial, the actions leading to it were intentional, and whether they served a medical purpose. To this end, we conducted a comprehensive morphological examination of the Chagyrskaya 64 molar, using traceological analysis, scanning electron microscopy, micro-CT analysis, and Raman spectroscopy. We also performed experimental studies on several modern *Homo* molars to test our hypotheses and substantiate our conclusions.

### Chagyrskaya cave: Stratigraphy, chronology and archaeology

**Fig 3:**
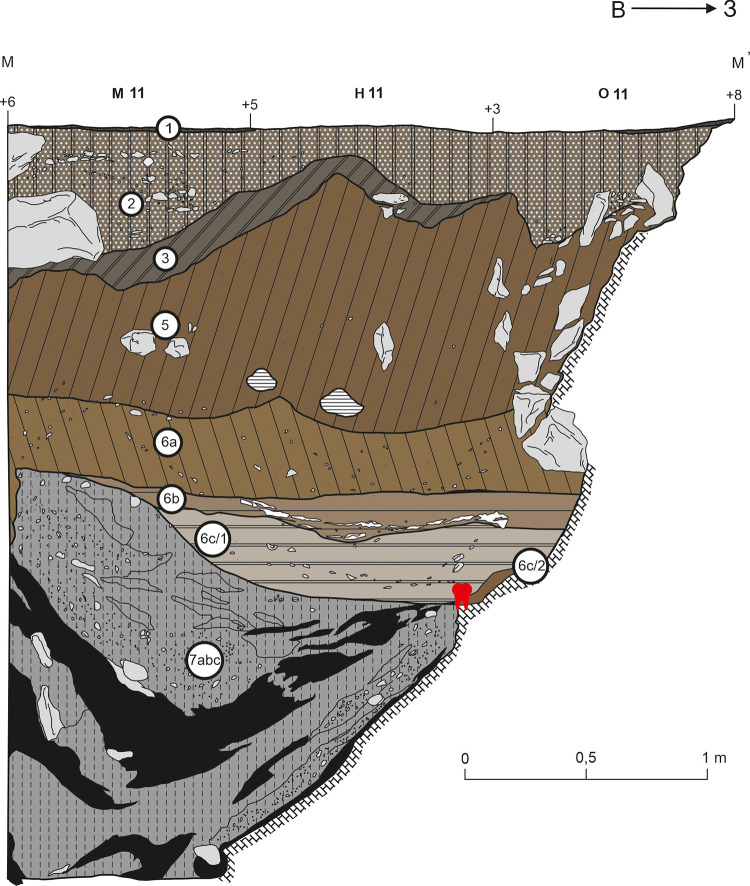
Discovery location of the Chagyrskaya 64 molar. Stratigraphic sequence (created in CorelDraw based on excavation data).

Chagyrskaya Cave (51°26′34.6″ N, 83°09′18.0″ E) is located on the left bank of the Charysh River that flows from the Tigirek Range in the northwestern foothills of the Altai Mountains (Fig 1: a, c). The cave, which has a northern exposure, is 19 m above the present river level at an elevation of 353 m above sea level. It comprises two chambers with a combined area of 130 m^2^. Systematic excavations at the site commenced in 2007 and, to date, approximately 50 m^2^ have been excavated to a depth of 3.5 m by two research teams (Fig 3).

Chagyrskaya Cave not only contains the most extensive assemblage of Neanderthal fossil remains in North Asia but also represents the easternmost known occurrence of the European Micoquian/Keilmessergruppen (KMG) archaeological technocomplex; a distinctive lithic tradition linked to Neanderthals who migrated into the Altai region approximately 70−60 ka [16].

Keilmessergruppen (KMG) constitutes a Middle Paleolithic variant documented in Central and Eastern Europe and the Altai region, associated with late Neanderthals and distinguished by the dominance of asymmetric backed bifacial knives (Keilmesser). The so-called “Eastern Micoquian” is regarded as a local KMG variant in the Crimea, parts of the Caucasus, and the Altai. KMG industries were distributed across this region between 160 and 35 ka BP. Their typological markers are asymmetric bifacial backed knives of diverse types (Keilmesser), generally produced using plano-convex bifacial technology [18]. The lithic inventory is characterized by a predominance of simple and convergent side-scrapers, along with retouched points [16]. The recorded variability of Eastern Micoquian assemblages reflects the distance and availability of lithic raw materials at a given site [19]. The production of bifacial Keilmesser involved the use of soft hammer percussion, which accounts for the numerous bone retouchers documented in the assemblages [20]. Beyond these, other categories of bone tools are also present. The technology for manufacturing plano-convex Keilmesser attests to the high degree of Neanderthal mobility: these artifacts were repeatedly utilized both as finished tools and as cores for flakes and new tool production, rendering them optimal for transport [21].

The Micoquian/KMG technocomplex has been documented at multiple sites across Central and Eastern Europe, the Caucasus, and Crimea, suggesting widespread cultural connections. These ties are further supported by genetic evidence derived from Neanderthal DNA sequenced at Chagyrskaya Cave, reinforcing the link between these geographically dispersed populations. Genomic analyses, including a high-coverage Neanderthal genome and nuclear DNA extracted from cave sediments, reveal that the Chagyrskaya Neanderthals exhibit stronger genetic affinities with late European Neanderthals than with those from the Middle Paleolithic layers of Denisova Cave (Denisova 5), also in southwest Siberia [22,23].

The stratigraphic sequence, sedimentological composition, and micromorphological characteristics of Chagyrskaya Cave’s sedimentary deposits have been comprehensively documented in previously published studies [16,24,25]. The stratigraphy reaches a maximum thickness of 3.5 meters, encompassing both Holocene and Pleistocene sediments (Figs 1: b; 3: a). These deposits have been systematically categorized into distinct layers (e.g., Layer 6), subunits (e.g., Subunit 6c), and sublayers (e.g., Sublayer 6c/1), with classifications based on lithological variations and erosional patterns. The stratigraphic units are further organized into three lithoseries [16], with Neanderthal remains specifically associated with Lithoseries II (Subseries IIa and IIb, respectively) (Fig 3: a).

Subseries IIa represents primary archaeological deposits in undisturbed sediments that preserve Middle Paleolithic artifacts and Neanderthal fossils in their original depositional setting (Layers 6c/2 and 6c/1, Figs 1: b; 3: a). In contrast, Subseries IIb exhibits comparable lithological properties but bears clear evidence of post-depositional colluvial transport, resulting in a secondary context for both cultural materials and fossil remains (Layers 6a and 6b).

The colluvial deposits in Subseries IIb appear to derive from the erosion and redeposition of material originating from both Subseries IIa and Lithoseries I, which were presumably located in deeper sections of the cave system. Micromorphological analysis indicates these sediments underwent limited transport and potential mixing, with the entire redepositional process constrained within a relatively brief temporal window. This interpretation is chronometrically supported by Optically Stimulated Luminescence (OSL) dating results, which constrain this depositional event to between 49–59 ka (95% probability interval) based on samples from Layers 6 and 5 [16]. The time span of deposition cannot be resolved more precisely at present. AMS radiocarbon (^14^C) and OSL ages obtained for samples collected from Layers 5 and 6 (Lithoseries II); full details are presented in Kolobova et al. [16].

The Chagyrskaya 64 molar was discovered almost on the contact with the cave’s bedrock substrate at the base of Layer 6c/2, partly preserved on a ledge along the western cave wall (Fig 3), reflecting an *in situ* “cave floor environment” [16] (Fig 1: b–d). The position of the specimen within the *in situ* Layer 6c/2 is hypothesized to have been a contributing factor to the preservation of this fragile molar. According to stratigraphic and micromorphological data, Layer 6c/2 represents the most intact stratigraphic unit in the cave, exhibiting negligible vertical translocation of archaeological material due to cryogenic processes. Nevertheless, considering the specimen’s location precisely at the interface of the tooth’s enclosing sedimentary layer and underlying bedrock, post-depositional movement can be precluded, thereby confirming its undisturbed *in situ* position. The stratigraphic position of the molar is associated with the earliest stage of the cave’s hominin occupation, ca. 59,000 years ago.

**Fig 4:**
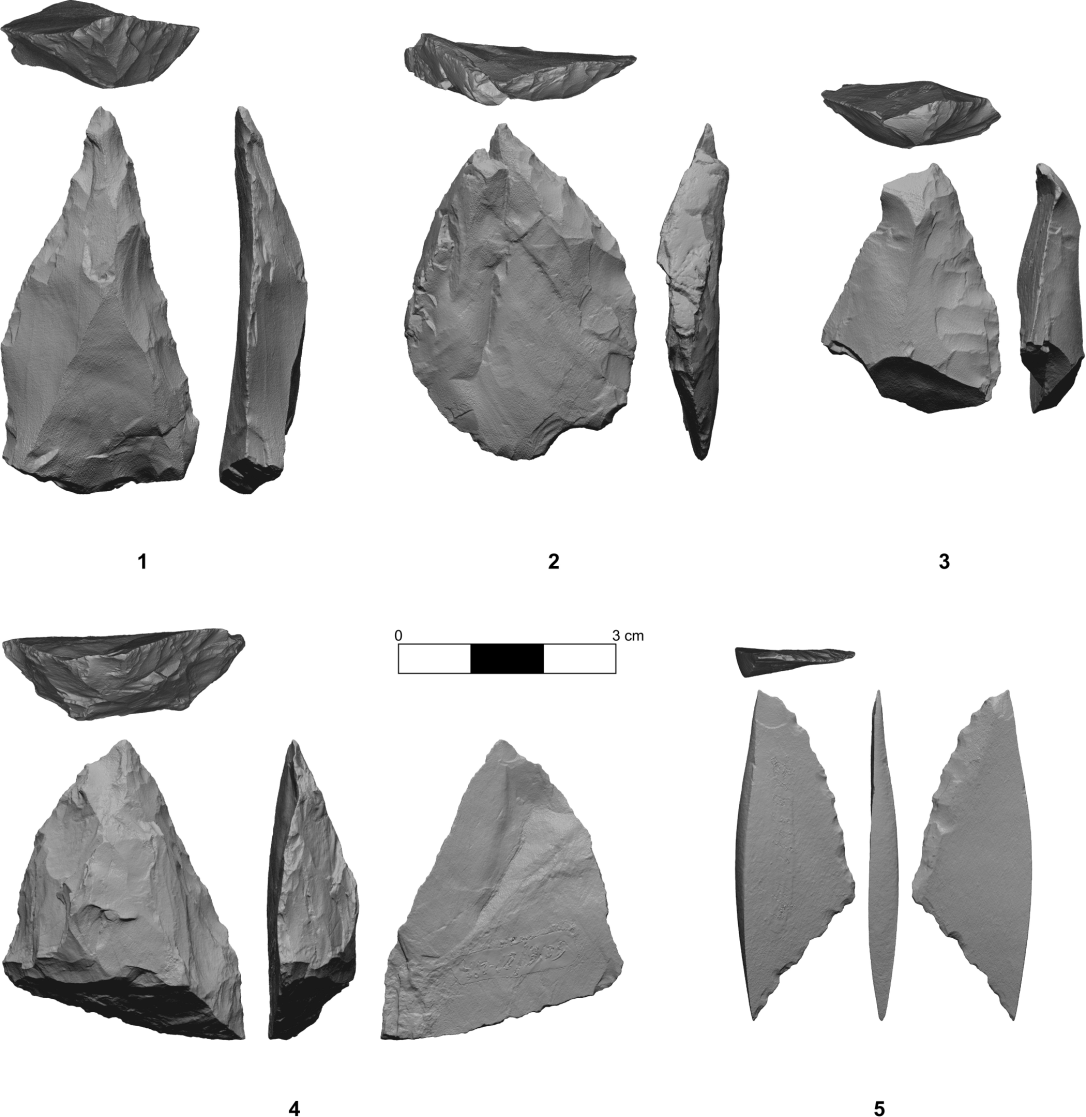
Retouched points (1, 4) and perforators (2, 3, 5) from Layer 6c/2 in Chagyrskaya Cave.

The lithic assemblage from Chagyrskaya Cave comprises the Sibiryachikha variant of the Altai Middle Paleolithic. Such assemblages are characterized by the predominance of radial flake cores and plano-convex bifaces including Keilmesser types (Bocksteinmesser and Klausennischemesser). Simple scrapers and trapezoidal and leaf-shaped convergent scrapers dominate the toolkit. The highest quality raw material in the Altai Middle Paleolithic, jasper, was used [16]. The toolkit contains several miniature retouched points and perforators that could potentially have been used for making holes and concavities in different objects close in size to the Chagyrskaya 64 molar (Fig 4).

## Materials and methods

### Description of the specimen

Chagyrskaya 64 is the lower second molar of an adult individual. The anatomical position of the tooth is ambiguous. In the initial publication describing this specimen [24], it was identified by B. Viola and coauthors as a left tooth, and this was consistent with our observations. However, Gicqueau et al. [26] have recently described Chagyrskaya 64 as a right tooth.

**Fig 5:**
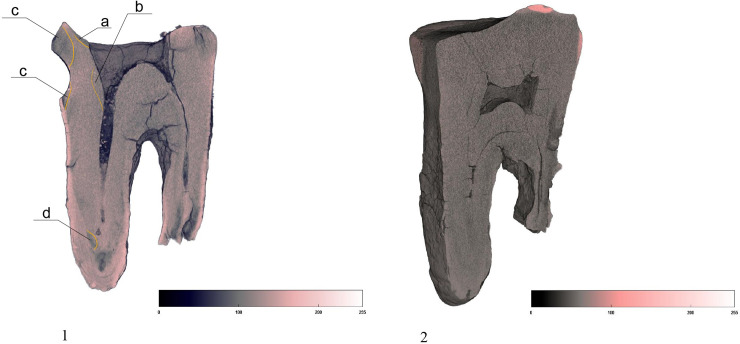
Comparison of Chagyrskaya 64 (1) and #1564 lower molar from Chagyrskaya Сave (2) dentine density distribution. Chagyrskaya 64 exhibits heterogeneous structure of dentine, with demineralized foci, marked by dark gray color and orange lines. #1564 shows homogenous dentine, without signs of demineralization. a, b, c, d. most intensively demineralized dentin areas. Light pink color of the Chagyrskaya 64 roots indicates hypercementosis.

The sample is almost complete, being preserved below its cervical margin. The crown is almost entirely absent, with no enamel preserved and substantial loss of coronal dentine due to attrition. The apical end of the mesial root is missing. This tooth exhibits severe pulp exposure without any secondary dentine formation (Stage 8) [27]. As a result, the functional occlusal surface of Chagyrskaya 64 is almost equivalent to a horizontal surface located near the cervix of the root. The root is mesotaurodont. The crown and roots also exhibit significant post-depositional cracking, and signs of hypercementosis are visible on the roots (Fig 5) Gicqueau et al. [26].

Other morphological features cannot be described due to extensive ante-mortem wear and a deep irregular concavity with smooth, rounded edges occupying most of the occlusal surface (Fig 2: 1).

Regarding the preservation state of Chagyrskaya 64, most of its surface (crown and roots) exhibits an uneven micro-relief and cracking (Fig 2: 1). However, no striations or other types of linear traces are present across the entire surface – indicating that post-depositional transport did not affect the tooth. All observed linear traces are confined to a specific area of the specimen, which is discussed in detail below.

The faunal remains from Layer 6c/2, primarily consisting of large herbivores such as bison and horse, are heavily fragmented (over 99%), yet they are characterized by good preservation. In more than 80% of the specimens, over half of the cortical surface is well preserved. The bones predominantly exhibit a low degree of weathering, most frequently corresponding to Stage 1 on the Behrensmeyer [28] scale, which points to their rapid incorporation into the sedimentary matrix. A small proportion of fragments show more advanced alteration (Stages 2–3). Evidence of root etching is observed on less than 1% of the specimens [29]. In comparison with the paleontological material, the human remains are similarly well preserved, with intact cortical surfaces and a consistently low degree of weathering (Stage 1). Given the inherent fragility of the Chagyrskaya 64 specimen, compounded by the presence of a large internal concavity, the fact that it was recovered intact serves as a strong indicator of its good taphonomic preservation within the layer. No alterations were detected on the tooth that could be associated with taphonomic processes.

### Traceological analysis

The entire surface of the Chagyrskaya 64 tooth was analyzed with an Olympus SZX7 microscope at low (8–56×) magnifications. The resulting observations were used to design and carry out experiments implemented to test different possibilities of occlusal surface modification. General morphology, macro- and micro-features on the experimental specimens were compared with those on the Chagyrskaya 64 molar, applying the full range of magnifications from low (8–56×) to high (100–500×). Micro-features, including wear, were analyzed using an Olympus BHMJ metallographic microscope at magnifications ranging from 100-500 × . The observations were recorded with a full-matrix Nikon D750 camera linked to the microscopes with Nikon Camera Control Pro2 software. Image recording was made using the stacking technique; resulting images were created in Helicon Focus 8 Pro software. A Gocator 3504 industrial profilometer was used to measure the identified marks.

### Experimental modeling

#### Experimental protocol and conditions.

To test our observations of the Chagyrskaya 64 tooth and verify our hypothesis, we attempted to experimentally replicate the process of intentional modification on the occlusal surface. For this purpose, we used three modern human teeth. Each step of the experimental modelling was designed for a specific objective: first, to assess the feasibility of using stone tools made from Chagyrskaya raw materials to create depressions through drilling or rotational action; second, to replicate the formation of two interconnected depressions in dentin through manual drilling with a lithic tool; and third, to expose the pulp chamber by drilling three interconnected depressions into the dentin. The objective of each successive step was adjusted in response to the outcomes of the preceding step.

Three well preserved modern *Homo sapiens* teeth served as experimental samples (see SI). Two of them exhibited either almost entirely worn occlusal enamel or complete enamel loss, a condition consistent with that observed on Chagyrskaya 64 (see SI, S3–S6 Figs). These teeth – an upper right second molar (RM^2^) and lower left third molar (LM_3_) – were obtained from undocumented Holocene archaeological collections curated by the Institute of Archaeology and Ethnography SB RAS in Novosibirsk. The third specimen, an upper left third molar (LM^3^) featuring a caries lesion on its crown enamel with no signs of previous dental treatment, was contributed by one of the authors.

We hypothesize that only lithic tools could be used for these operations, as neither bone, wood, nor other materials available to Neanderthals would have been capable of modifying the tooth’s structure. This is supported by earlier experimental studies conducted by Oxilia et al. and Coppa et al. [12,30], which conclusively demonstrated that creating a concavity in dental tissue using bone or wooden tools is highly impractical. In the case of Chagyrskaya 64, the concavity is notably deep, extending to the pulp chamber floor. Given this depth, the use of bone or wooden tools is deemed improbable due to their limited effectiveness in such invasive modifications. Instead, we propose that the procedure was performed using a small lithic tool with a narrow tip.

**Fig 6:**
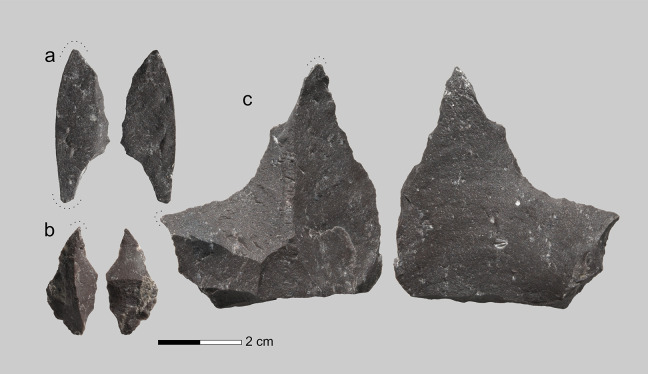
Experimental tools fabricated from local jasperoid raw material. Dotted lines show the active parts of the tools. a–c. perforators.

Chagyrskaya includes a variety of tools (Fig 4) that meet the required size and morphological criteria (tiny with an elongated, thin point). The most likely candidates for such a task are typologically classified as small points or perforators with minimum dimensions of 29 mm in length and 19 mm in width. To test this hypothesis, we replicated the concavity on experimental human teeth using perforators manufactured from local jasper found in the vicinity of Chagyrskaya Cave (Fig 6).

To replicate the procedure under biologically plausible conditions, all experiments were conducted with the addition of a small amount of water, simulating the moist environment of the human oral cavity. Each experimental tooth was secured in a cork base – embedded root-first – to ensure optimal stability during the process (S1 Fig). All experiments were conducted by one individual experienced in Palaeolithic technologies, specifically stone knapping, as well as the production and use of lithic and bone tools.

#### Experimental modeling limitations and assumptions.

Limitations of the experimental modeling include morphological differences between the lower molars of modern *Homo sapiens* and *Homo neanderthalensis*. Of critical importance are, first, interspecies variations in enamel and dentin thickness and, second, differences in the position of the pulp chamber floor and root bifurcation point relative to the cervical margin of the tooth.

Neanderthals are characterized by relatively thinner enamel compared to *Homo sapiens*, distributed over a larger volume of coronal dentin [31–34]. Consequently, during our experiments, it was necessary to account for the fact that the initial penetration into the dentin layer may have required less time and effort for the Chagyrskaya 64 tooth, presumably damaged by caries, than was needed to replicate these manipulations on experimental specimens.

Additionally, the Chagyrskaya 64 molar exhibits certain taurodontic features, including an enlarged pulp chamber and an apically displaced root bifurcation point [26]. This results in a greater volume of affected tissue compared to what would be expected if the tooth derived from *Homo sapiens.*

The absence of identical Neanderthal molars in the present day obviously precludes the experimental replication of concavities with perfectly matching morphology and dimensions to those observed on the Chagyrskaya 64 molar. A further limitation stems from the taphonomic state of all known Neanderthal specimens – including their mineralization, dehydration, and other diagenetic alterations resulting from their great antiquity. To reproduce the modification process, a tooth in a more or less fresh condition would be required. Finally, any Middle Paleolithic Neanderthal tooth constitutes irreplaceable evidence of hominin evolution, rendering any procedure that risks irreversible damage ethically impermissible. For these reasons, direct experimental studies on original Neanderthal teeth were not undertaken.

Nevertheless, the processes involved in creating depressions – along with the macro- and microstructural characteristics of the modified concavity walls – can be convincingly reconstructed and analyzed with *Homo sapiens* molars (S1–S6 Figs).

Another limitation of our experimental modeling lies in the difficulty of precisely replicating the oral environment. In vivo, the molar would have been situated in a confined space within the mouth, surrounded by other teeth, and access to it was likely more restricted than what could be reproduced experimentally.

However, our objective was not to replicate the entire process, but to determine whether the observed concavity shape and associated surface modifications could be produced on dentin. For this purpose, the limitations discussed are not critical.

### Scanning electron microscopy

Surface morphology of the tooth was analyzed using a Hitachi S-3400 N scanning electron microscope (SEM). The range of magnifications is from 7 to 300,000 × . The accelerating voltage was 20 keV. No conductive coating was applied to the sample, a low vacuum mode (40 Pa) was used to remove the charge. Images were recorded using a backscattered electron detector (compo mode).

### Micro-CT analysis

Micro-computed tomography (micro-CT) scanning was carried out at the Research Centre for X-ray Diffraction Studies, Saint Petersburg State University, St. Petersburg, Russia using a Bruker SkyScan-1172 micro-CT with a tube voltage of 100 kW, and 100 mA with a 0.5mms aluminum filter. The rotation step was 0.25°, 9.65 mM resolution. Image slices were reconstructed in NRecon (Bruker-microCT, Kontich, Belgium). A 3D digital model of the tooth was created using the CTAn (Bruker-microCT, Kontich, Belgium) and dental tissues were virtually separated. Visualization of the digitized model was carried out using CTVox (Bruker-microCT, Kontich, Belgium).

### Raman spectroscopic analysis

The tooth surface was examined by Raman spectroscopy. A M532/785 microscope (Spektr-M) was used: spectral range 100−4000 cm − 1, slit aperture 20 microns. Nd:YAG laser with a wavelength of 532 nm (maximum power 50 MW) was used. The tooth surface was analyzed at 14 individual points. Additionally, to verify measurements, the surface of experimental specimen #3 was also studied. Each was analyzed at two points (e.g., six spectra were obtained). The analyses were carried out in an air environment, no cleansing was performed, and the time for spectrum acquisition was 180–360 seconds. Spectra with peaks formed, which indicate bursts of radiation intensity characterized by a definite Raman shift (in cm-1) [35]. The study was carried out using instruments of the “Cenozoic Geochronology” Core Facilities Center at the Institute of Archaeology and Ethnography SB RAS (Novosibirsk, Russia).

No permits were required for the study described, which complied with all relevant regulations.

## Results

### Ante-mortem modification on Chagyrskaya 64

**Fig 7:**
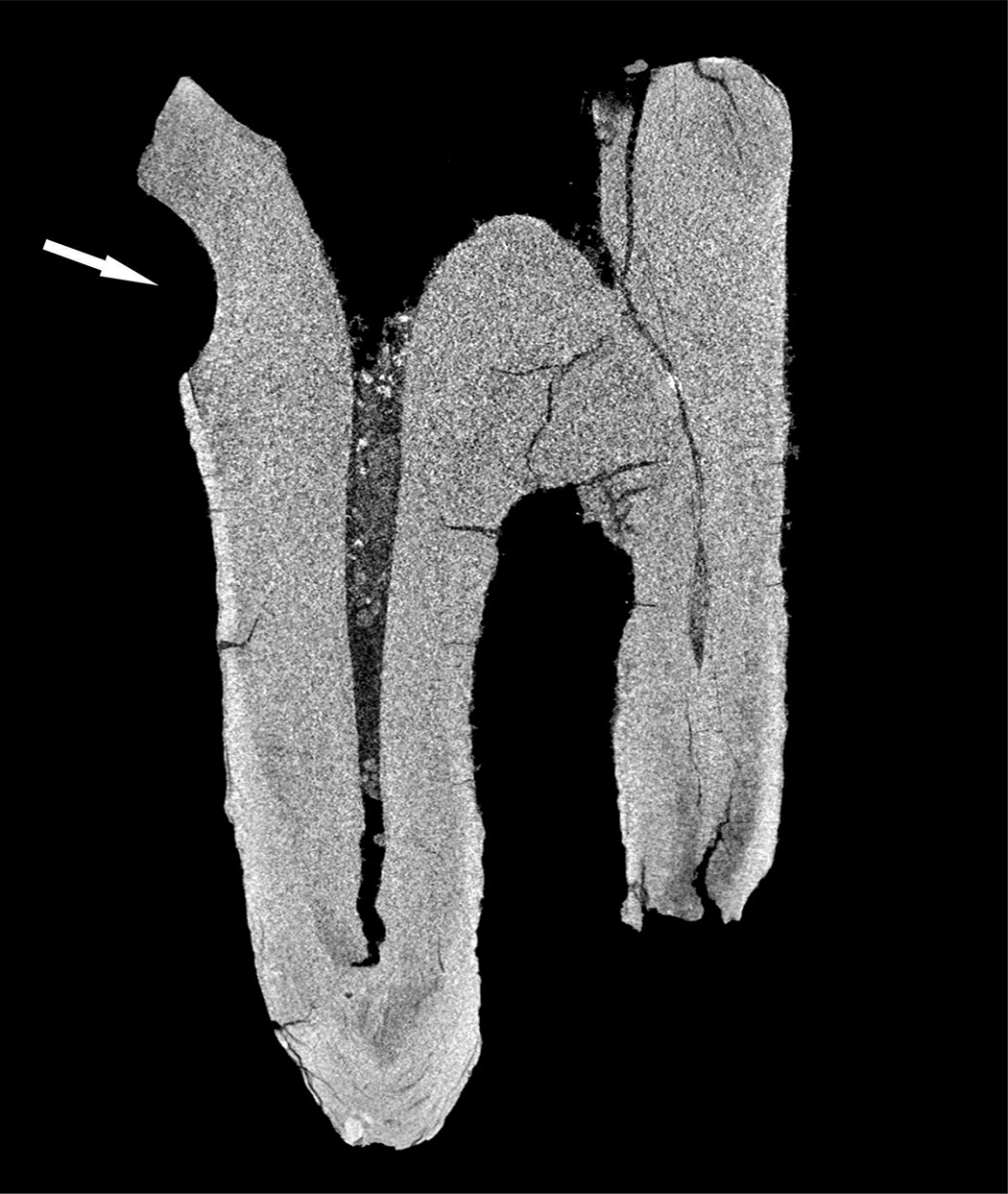
Chagyrskaya 64, CT-scan. Arrow indicates a toothpick groove.

**Fig 8:**
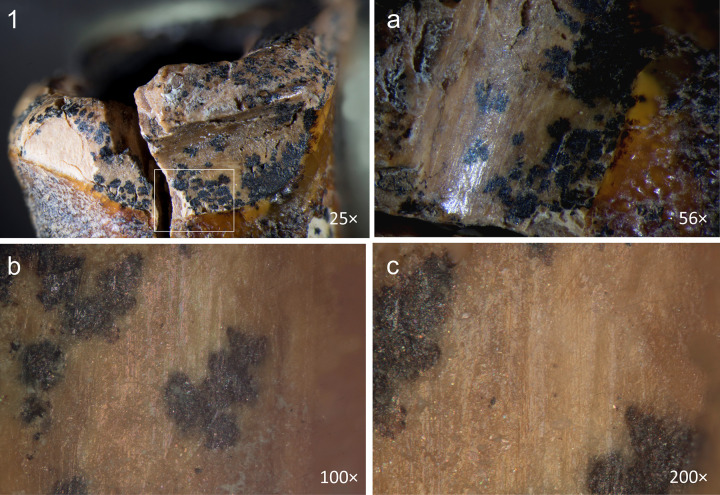
Chagyrskaya 64, toothpicking groove and linear traces on the distal interproximal surface. 1 macro-photo of the groove; a–c. macro- and micro-photos of linear striations.

The Chagyrskaya 64 molar (Fig 2) is that of an adult Neanderthal individual. A pronounced interproximal groove, is present on the distal aspect at the cervical area (Figs 7 and 8). The macroscopic characteristics of the groove – its regular margins, gently sloping polished walls, and specific location – combined with the microscopic evidence of multiple microstriations parallel or subparallel to the enamel-dentine junction, concentrated only in the groove, indicate that it is typical of toothpicking (Fig 8). The described features are classified by Estalrrich et al. 2017 as Stage 4 of toothpick use-wear characterized as well-developed (1.5–2.8 mm in width) and macroscopically visible [36]. This morphology indicates consistent toothpick use, repeatedly documented in numerous groups of archaic and near-modern *Homo* [14,37–40] (S7 Fig).

**Fig 9:**
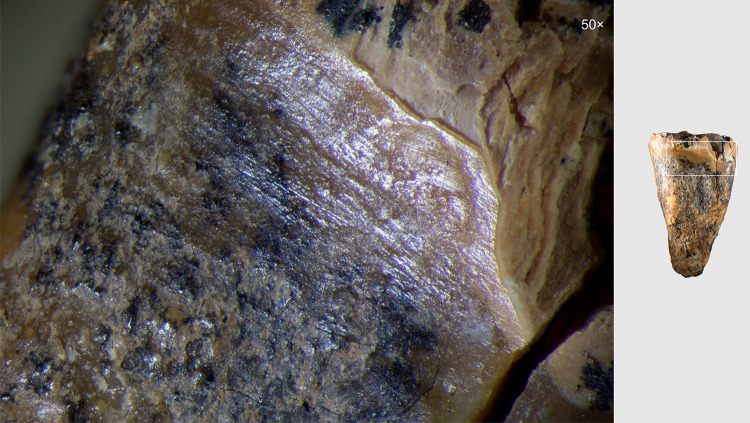
Chagyrskaya 64, linear traces of toothpicking on the mesial approximal surface.

The mesial interproximal surface of Chagyrskaya 64 features a circumscribed polished area bearing linear microstriations, oriented buccolingually in a parallel to subparallel fashion (Fig 9). These characteristics are diagnostic of a nascent stage of groove formation, resulting from the same mechanical process. This wear pattern is well-established in the literature as indicative of initial toothpick use, Stage 2 according to Estalrrich et al. [36].

The absence of microstriations on the surrounding uneven microrelief, the tight localization of the polish to the interproximal surface, and the lack of postdepositional transport in the containing Layer (6c/2) [16] all confirm that the modification of the interproximal area on Chagyrskaya 64 is not taphonomic. Instead, it is attributed to ***antemortem toothpicking activity***.

**Fig 10:**
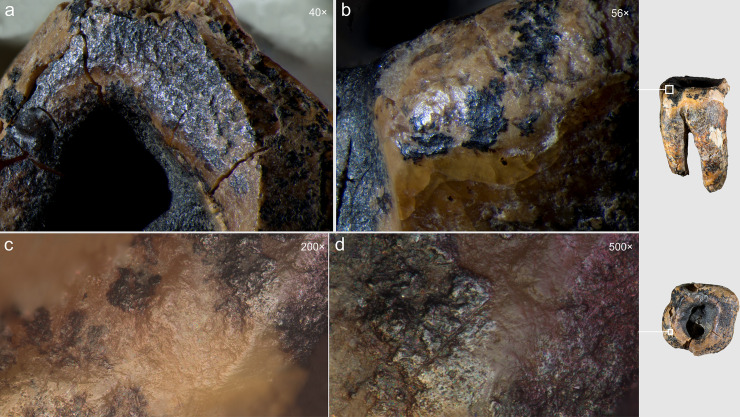
Chagyrskaya 64, unmodified crown sections with characteristic polish from ante-mortem wear. a, b. macro-photos; c, d. micro-photos.

The occlusal enamel is completely worn away. Unmodified areas of the crown’s masticatory surface retain evidence of ante-mortem wear, manifest as characteristic ***smoothed and polished areas*** (Fig 10), predominantly preserved as isolated patches.

A ***large central concavity*** with 4,2 mm length, 2,8 mm width and 2,6 mm maximal depth is present on the molar’s occlusal surface, comprising three interconnected and partially overlapping depressions (Figs 2: a; 11). Micro-CT analysis (Fig 12) revealed that this concavity occupies the entire pulp chamber volume, with transverse expansion in the mid-crown region. The distal margin of the concavity exhibits a more gradual slope compared to the mesial margin. The depth of the concavity varies across different sections of the tooth. In the central portion, it is confined by the floor of the pulp chamber, while extending into the pulp horns in both distal and mesial sections.

**Fig 11:**
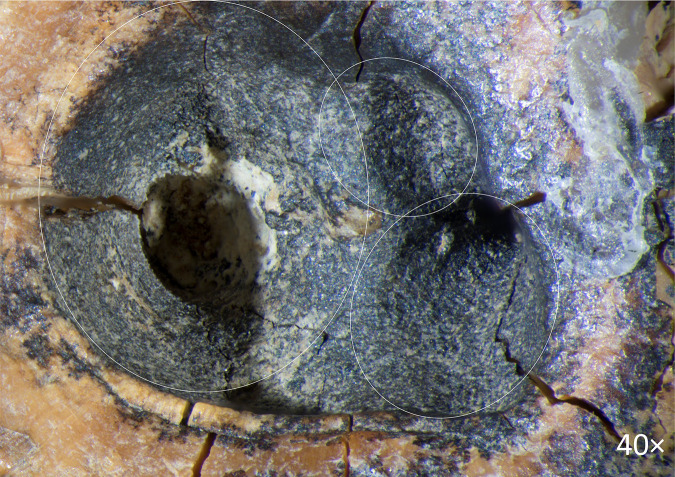
Chagyrskaya 64, three recesses in the occlusal surface of the crown (macro-photo).

**Fig 12:**
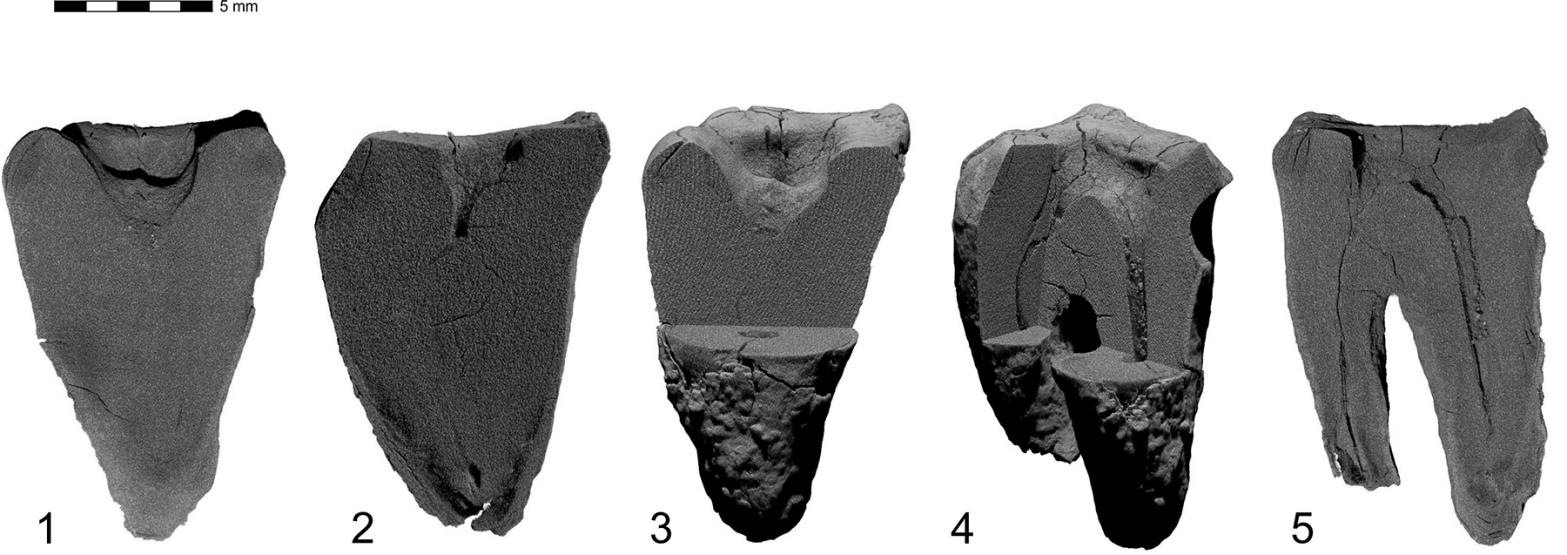
Results of CT-scan of the Chagyrskaya 64 molar. Morphology of the concavity is shown in five projections, from left to right: mesial (1) distal (2, 3), disto-buccal (4), buccal (5).

Theoretically, there could be several reasons for the appearance of concavities on the occlusal surface of a molar. The main ones are ante-mortem wear (abrasion and attrition) [41], dental trauma, and caries.

Typical features of dental trauma are sharp edges of the enamel or dentine defects with cracking and no remarkable alteration of the density in the surrounding tissues. The edges of the Chagyrskaya 64 occlusal concavity are rounded and smooth, which is not typical of enamel fracture edges. Therefore, the depression is unlikely to be of traumatic origin.

The effect of dental wear in the case of Chagyrskaya 64 may be significant due to the advanced age of the individual [26]. However, as several studies have described, occlusal wear can lead to pulp exposure only in cases of extremely rapid pathological attrition caused by abnormal use or positioning of the teeth. If attrition occurs at a rate faster than the formation of secondary dentin, the pulp cavity will be exposed, creating the potential for infection of the pulp and supporting alveolar bone [42].

Carious lesions are the result of chemical demineralization of dental tissue and its decomposition under the influence of acids released during the fermentation of carbohydrates by acid-forming cariogenic bacteria. The latter include lactobacilli and staphylococci, among which *S. mutans* is one of the most active, as well as some others [43].

The main methods for diagnosing caries and differentiating it from pathological attrition are visual observation, histological and X-ray examination, and CT analysis. The use of CT has increased widely over the last decade [44,45] as a non-destructive method suitable for both in-vivo and in-vitro analysis. The main criterion for CT diagnosis of caries is progressive demineralization of enamel and dentin [46,47], determined according to the Downer scale [48]. According to this scale, carious lesions correspond to five degrees of demineralization of dental tissues:

Grade 1. No enamel demineralization or a narrow surface zone of opacity.
Grade 2. Enamel demineralization limited to the outer 50% of the enamel layer.
Grade 3. Demineralization involving the inner 50% of the enamel up to the dentino-enamel junction.
Grade 4. Demineralization involving the outer 50% of the dentine
Grade 5. Demineralization involving the inner 50% of the dentine [48].

To assess whether the occlusal concavity of Chagyrskaya 64 was the result of pathological attrition or a carious lesion, we compared micro-CT scans of Chagyrskaya 64 with four upper and three lower molars from the Chagyrskaya Cave in terms of dental tissue demineralization and the form of the occlusal surface.

The Chagyrskaya 64 molar shows an absence of secondary dentine and a significant amount of demineralized primary dentin, marked in dark gray (Fig 5). The largest foci are localized in the distal part of the crown: on the occlusal surface around the central concavity (Fig 5: a); along the distal root canal in the region of its connection with the pulp chamber (Fig 5: b); on the upper and lower edges of the distal tooth-pick groove (Fig 5: c); and in the apical parts of the roots (Fig 5: d). Demineralization affects both the inner and outer dentin, which is consistent with deep caries, grade 4 or 5 [46–48].

Such conditions were not observed in the comparative samples (Fig 5). For instance, molar #1564, which occupies the same stratigraphic position as Chagyrskaya 64, demonstrates a uniform structure of dentine without foci of demineralization, despite the intense wear of the occlusal enamel (Fig 5).

The absence of secondary dentine in Chagyrskaya 64 is unusual for heavily worn teeth. Also atypical is the occlusal surface profile, as the occlusal surfaces of all comparative lower molars, such as #1564 (Fig 5), are flat and lack any concavities. The shape of the concavity in question differs from the normal pulp chamber morphology due to the widening in its upper part. This provides another argument against the hypothesis that the concavity formed as a result of dental wear, since the latter can only expose the pulp chamber but not widen it.

Therefore, we conclude that dental attrition is unlikely to be the cause of the occlusal surface concavity in Chagyrskaya 64. Dental caries represents a more plausible and probable explanation. The localization of demineralized foci indicates that the molar exhibited at least two primary carious lesions: one on the occlusal surface and another in the cervical area, around the distal tooth-picking groove. One of the most prominent foci of demineralization is located in the upper part of this groove (Fig 5). The absence of secondary dentine and the widening of the upper part of the concavity suggest the intentional removal of some dental tissue.

The depositional environment itself argues against a taphonomic explanation. The absence of postdepositional disturbance in Layer 6c/2 [16] precludes sedimentary processes as the agent of this occlusal modification. Moreover, even setting the context aside, there is no known geological mechanism that could carve a deep concavity into hyperdense dental tissue.

Excluding the alternative interpretations considered here, and taking into account the unusual form of the exposed concavity with secondary dentine absent in this heavily worn tooth, we hypothesize that the concavity is the result of artificial modification of a carious lesion. This is confirmed by both the concavity’s overall configuration and proportions, which are very similar to other known examples of human invasive mitigations of caries lesions [12,30,49,50]. The morphology of the depression suggests intensive dentin removal to gain access to the pulp chamber (Figs 2: 1; 12).

**Fig 13:**
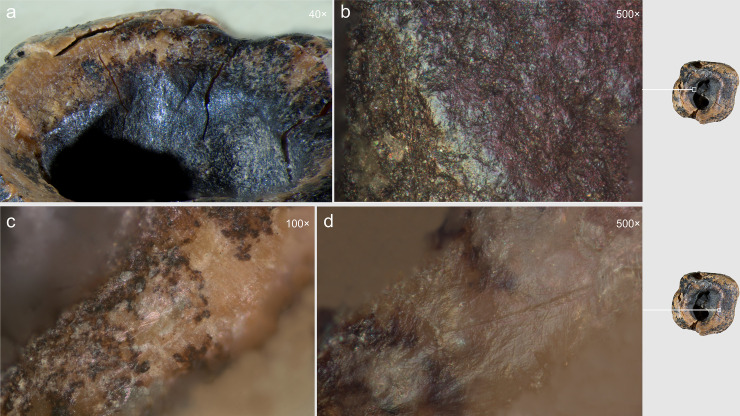
Chagyrskaya 64 ante-mortem modification of the concavity. a, b. smoothing and polish on the walls; c, d. polish on the edge.

On the buccal side of the tooth, at the edge of one of the depressions, ***two parallel step-like furrows*** were observed (Fig 2: b). These microgrooves have nearly V-shaped profiles, with a maximum width of 0.294 mm and a depth of 0.076 mm (Fig 2: c). The outer wall of the grooves is more gently sloping compared to the wall facing the central part of the crown (Fig 2: 1, a). In certain areas of the concavity, these microgrooves are overlain by ***areas of ante-mortem polish*** (Fig 13). We hypothesize that this feature, rather than forming through natural attrition, was created by intentional dentin removal.

Furthermore, the edges of the concavity along the walls exhibit, not sharp, but markedly ***smoothed and even rounded contours***. These areas also display distinct polish, which is more pronounced than that observed on the concavity walls themselves (Fig 13: a, c, d). This indicates that the crown tissues were removed during the individual’s lifetime and that the tooth remained in active use following the dental intervention. The polish is less pronounced than that observed on the occlusal surface (Fig 10), as the contact between the concavity walls and food particles or raw material being processed with the teeth (e.g., plant fibers, animal sinew) occurred during a shorter duration.

Additionally, following the hypothesis of the human origin of the concavity, it may have been intermittently filled with wax, tree sap, bitumen or another antiseptic and potentially analgesic material, which could also account for the slightly different pattern of ante-mortem wear observed within the concavity [51–55]. To test this hypothesis, we performed Raman spectroscopy.

**Fig 14:**
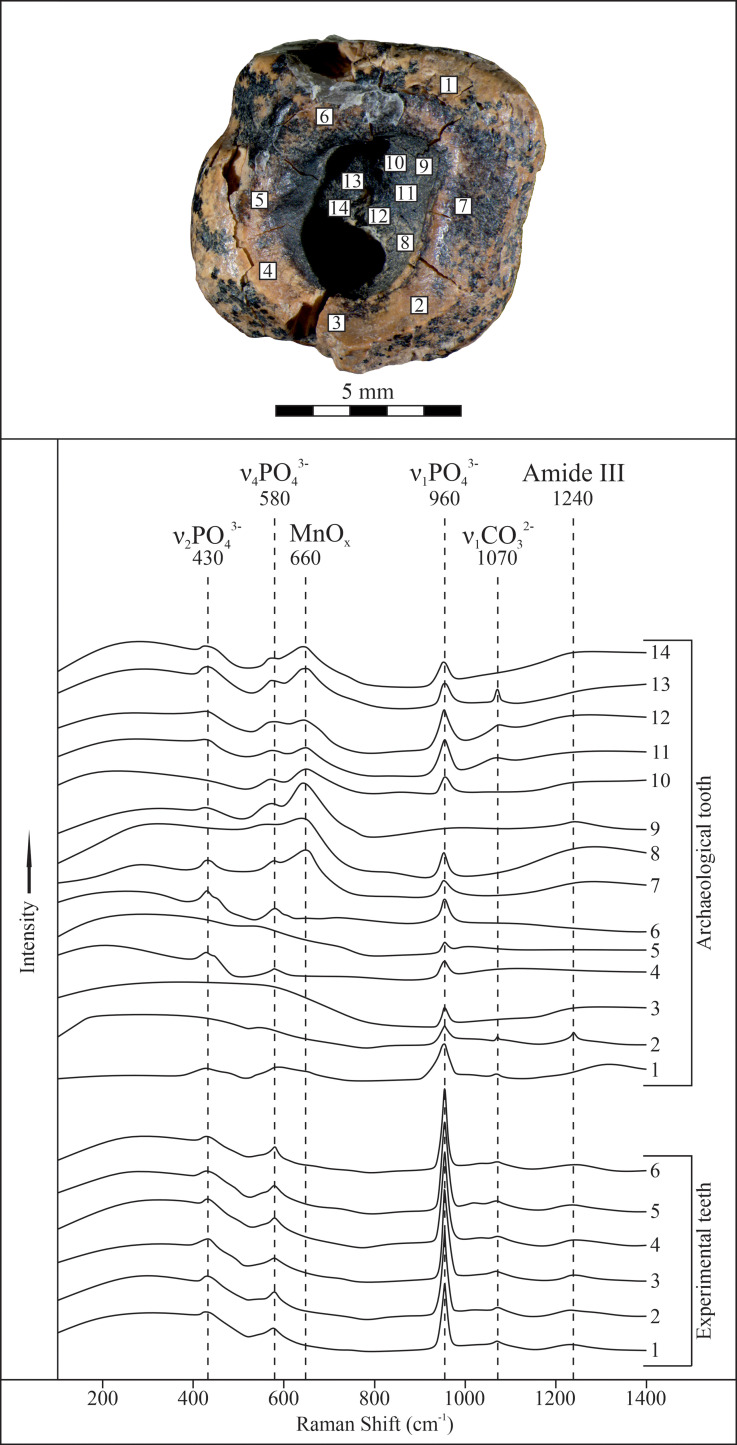
Results of Raman spectroscopic analyses of the tooth surface. Analysis points at the top; Raman spectra of the tooth and experimental samples at the bottom.

A group of phosphate (PO_4_) peaks of 430, 580, and 960 cm-1 was identified (Fig 14). In some cases, a zinc carbonate (ZnCO_3_) peak of 1070 cm-1 and an amide III (nitrogen compound) peak of 1240 cm-1 were recorded [56–58]. The Raman spectra of the dark surface (sections 8−14) are characterized by the presence of an intense manganese oxide (MnOx) peak of 660 cm-1 [59]. Manganese is a part of dental dentin and, when the tooth surface is destroyed due to taphonomic processes, black MnOx is formed [60]. Thus, multiple Raman spectroscopic analyses revealed only dentin and ***did not detect any other residues*** (Fig 14).

**Fig 15:**
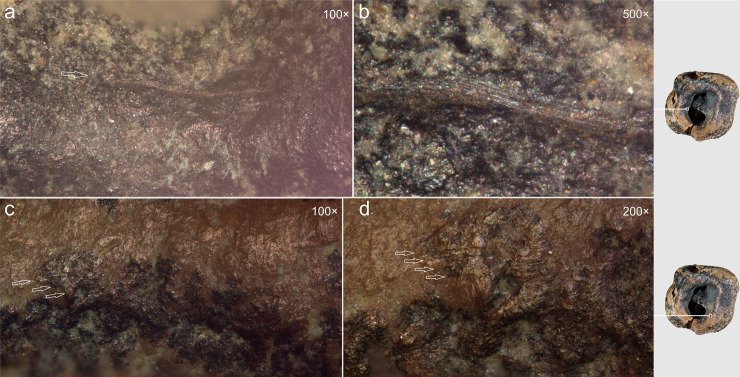
Chagyrskaya 64, microscopic traces indicative of rotation technique on the interior walls of the concavity. Horizontally oriented linear traces run parallel to the concavity edges at two distinct locations (a–b and c–d). a–d. micro-photos.

Optical microscopic examination revealed that, in addition to the major microgrooves along the concavity’s margins, ***fine linear striations*** were preserved on the less worn areas, visible at magnifications of 100 × , 200 × , and 500× (Fig 15). These microstriations are oriented parallel to the concavity walls, which could result from the rotational motion of the tool’s working edge. They exhibit a distinctive “ridged” (corrugated) base: each microstriation consists of a series of parallel, extremely fine scratches.

The linear microstriations and microgrooves exhibit smoothing (Fig 15: c, d). This observation explains, on the one hand, why traces associated with the initial formation of the concavity (dentin removal) are less distinct. On the other hand, the pronounced smoothing of the concavity walls indicates ante-mortem wear occurring after the depressions had already formed, which effectively rules out a taphonomic aetiology.

### Experimental verification results

The morphology and traceological features observed on the interior walls and edges of the concavity in the occlusal surface of the Neanderthal Chagyrskaya 64 tooth suggest they resulted from intentional drilling or scraping activities aimed at removing carious lesions. To further evaluate the hypothesis of human agency in the formation of the concavity, experimental modeling was undertaken.

Two technical variants were employed: scraping and rotating (hand drilling). The first method demonstrated limited efficacy during the initial stages of concavity formation, as it proved challenging to concentrate force on a single point, resulting in isolated striations that failed to produce a pronounced depression.

The second technique – using the point as a hand drill or perforator – enabled the disruption of the tooth surface integrity within the first few seconds. As a result, this method was employed in all subsequent tests.

To access the pulp chamber, concavities were created on the occlusal surface of the experimental teeth. This was achieved through manual rotation using small lithic tools with narrow, pointed tips (Fig 6). This experimental procedure was performed on three modern human molar specimens (see SI).

Each experimental session lasted less than one hour: experiment #1 took just over 5 minutes, experiment #2 approximately 35 minutes, and experiment #3 roughly 50 minutes (penetration to the pulp chamber). Even considering that the tooth was situated in the mouth, surrounded by other teeth in a narrow space and with complicated access to the damaged occlusal surface, the efficiency of the tool and technique remain crucial factors. We believe that even if the time required for pulp penetration was doubled due to restricted access, the individual would have had little choice: remove the dentin or face potentially fatal consequences.

The processing marks observed on the concavity walls of experimental tooth #1 (at both macro- and micro-levels) manifest as distinctly pronounced, relatively deep linear striations, parallel to concavity margins (S2 Fig: d, e). The micro-relief of these linear striations exhibits clearly defined contours (S2 Fig: f, g) due to contact of enamel with solid material – jasper. The absence of enamel on the Chagyrskaya 64 tooth explains why comparable marks are not observed there.

Experimental sample #2, with an almost entirely worn occlusal enamel surface, demonstrated that the dentin area showed markedly more delicate traces: much less pronounced, appearing more superficial and much smaller. They are difficult to perceive at the macroscopic level (S3 Fig: c–e).

Experiment #3 revealed that when perforation occurs at the base of a dentinal concavity, pre-existing cracks may propagate, resulting in the fracture of a larger coronary dentin fragment. This provides greater access to the pulp chamber than could be achieved through drilling alone (S4 Fig: d).

Macroscopic and microscopic analyses of the experimental teeth revealed distinctive linear striations covering the dentin. These are primarily superficial, parallel to subparallel, and exhibit a concentric arrangement that follows the contours of the concavity. Near the base, some microstriations display different orientations – including diagonal and even vertical patterns (S5 Fig: c, d) – likely resulting from minor adjustments in tool kinematics, specifically the introduction of partial scraping motions.

This series of experiments demonstrates that the same type of occlusal concavity can be reproduced on a human molar through the application of a small lithic tool in a drilling or rotating motion.

## Discussion

Experimental results (see S1 Text) confirmed that the concavity formation for pulp chamber access is not only feasible but can be effectively achieved through unassisted manual drilling/rotational motions using a small lithic tool with a narrow point (perforator) manufactured from jasperoid raw material (Fig 6). While scraping proves suboptimal during initial concavity preparation stages, our experiments indicate this technique becomes functionally applicable – and potentially more efficacious than drilling – during the terminal operative phase immediately preceding pulp chamber penetration. The morphology of the concavities – both in the Chagyrskaya 64 molar and experimental teeth – show comparable features, exhibiting similar sub-rounded contours (Figs 2: 1; 11 and 12; S2–S5 Figs).

Due to the inability to procure experimental specimens anatomically identical to the archaeological sample, structural differences between *Homo sapiens* and *Homo neanderthalensis* molars prevented exact replication of the concavity formation processes, particularly with respect to perforation techniques and pulp chamber scraping. Nevertheless, a critical observation emerged concerning potential perforation mechanisms during terminal drilling phases. As demonstrated in our final experiment, #3, the thin dentin layer overlying the pulp chamber is extremely fragile and prone to spalling when concavity depth approaches critical limits (S5 Fig). This phenomenon may explain the perforation pattern observed on the Chagyrskaya 64 molar and the paucity of observable tool marks on it. Given the evident carious degradation in this dental area, the dentin likely presented even greater porosity – potentially accelerating pulp chamber exposure compared to our experimental conditions. It would preclude the formation of characteristic drilling or scraping traces in this specific area.

**Fig 16:**
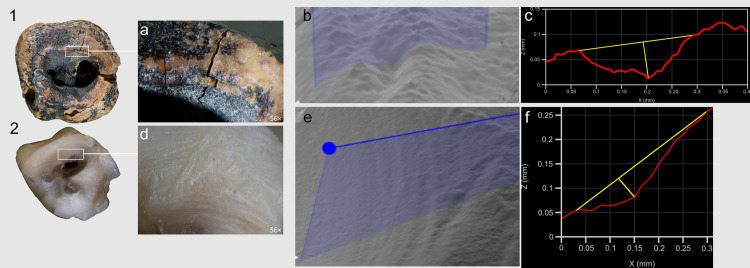
Comparison of the linear traces forming concavities on Chagyrskaya 64 and experimental tooth #3. 1, 2 top views; a, d. macro-photos of grooves along the edges of the concavity walls; b, e. profile measurement points; c, f. profile measurements.

A critical observation emerged from the experimental modeling and analysis of macro- and micro-traces on dentin surfaces (Fig 16). Macroscopic traces appear significantly less pronounced on the dentin surface of experimental specimens compared to enamel surfaces, explaining the near absence of visible macro-scale processing evidence on the Chagyrskaya 64 molar’s concavity walls. Micro-traces associated with dentin processing manifest as predominantly fine, superficial parallel and subparallel linear striations, interspersed with occasional deeper individual grooves (Fig 16: d). As a rule, these grooves have a corrugated bottom and well-defined edges.

Comparative analysis of micro-traces observed on the dentinal walls of experimental teeth versus those present on the Chagyrskaya 64 molar (Fig 16) revealed that post-operative wear and smoothing significantly altered the microtopography (Fig 13), resulting in the loss of most processing marks. Nevertheless, isolated evidence of rotating with lithic tools produced during initial concavity formation remains detectable (Fig 15).

Our experiments revealed that the grooves along the concavity’s edge resulted from circumferential movements of a small notch on the tool’s working part, which created the stepped topography during the drilling process (S6 Fig: a, b). Due to ante-mortem wear and post-mortem damage, the depth of the major grooves on the Chagyrskaya 64 molar (0.076 mm) is less pronounced than those produced experimentally (0.452 mm). Additionally, the experimental linear macrotraces exhibit clearer outlines than their archaeological counterpart. The groove profiles in both cases display a similar V-shaped morphology, with the outer wall being more gently sloping than the inner one (Figs 2: b; 16) The width of the experimental groove (0.249 mm) closely matches that observed on the archaeological tooth (0.294 mm).

**Fig 17:**
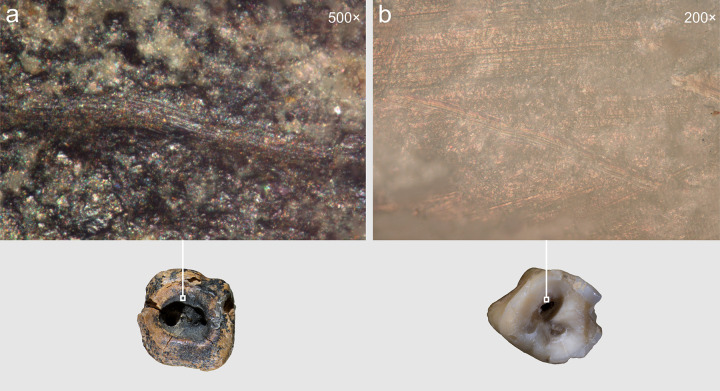
Microscopic evidence of concavity formation through drilling/rotating. Horizontal linear striations aligned parallel to the concavity’s edges are visible on the concavity walls. Comparative illustrations of these linear striations. **a.** Chagyrskaya 64 Neanderthal molar; b. experimental modern *H. sapiens* specimen #3.

Experimental drilling produced linear macro- and microtraces that matched those on the Chagyrskaya 64 molar in both characteristics and orientation (Figs 16 and 17).

As noted earlier, the concavity comprises three partially overlapping depressions, suggesting formation in at least three distinct stages (Figs 11 and 12: 1, 3). Whether these depressions represent separate temporal episodes remains unclear, as no differences in their preservation states are discernible. There is likewise no evidence to confirm that they result from multiple attempts at caries treatment, although this possibility cannot be excluded.

The third experiment attempted to replicate this configuration (see SI; S4 Fig: b, c). Our observations indicate that only two penetration points were necessary to open the pulp chamber. Moreover, as the two depressions providing canal access were enlarged, developing the third became progressively more challenging, and it was ultimately partially superimposed. Extrapolating to Chagyrskaya 64, we infer that the smallest depression represents an early stage of concavity formation, while the two deeper depressions that reached the canals were expanded after it had already been made (Fig 12: 1, 3). Accordingly, we suggest two possibilities: the third depression was either intended to treat a limited carious area or was an error, as the technique lacked perfect precision.

**Fig 18:**
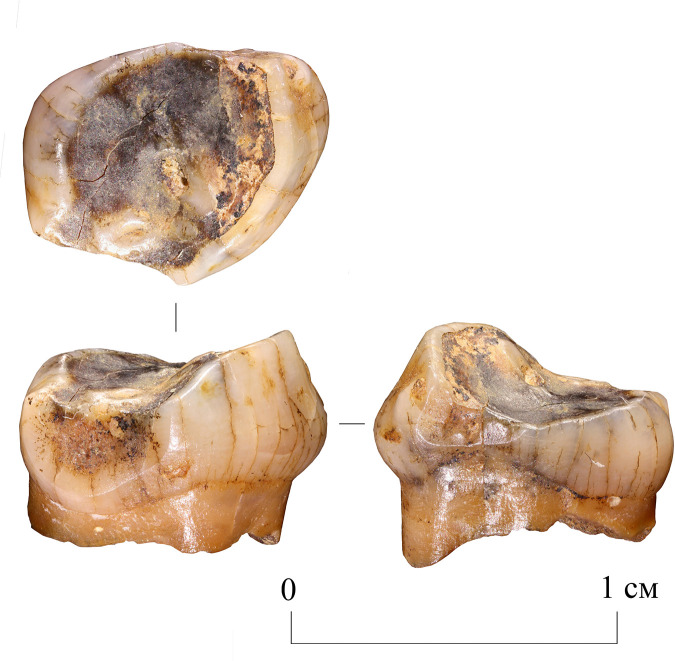
Chagyrskaya 18. Occlusal, distal and mesial views.

**Fig 19:**
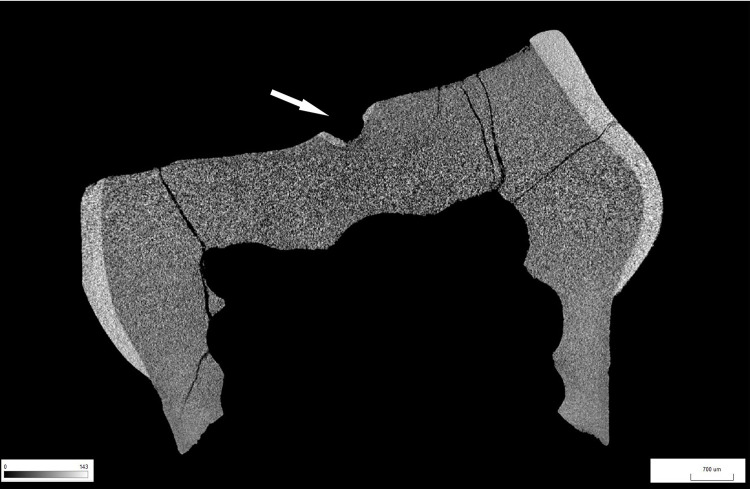
Chagyrskaya 18 micro-CT image. Carious lesion on the occlusal surface of the molar. Transverse section. The arrow points to the area of damaged enamel.

The results of micro-CT analysis and the morphology and location of the concavity on the Chagyrskaya 64 molar provide reasonable grounds to suggest that its formation was related to the intentional invasive treatment of dental caries. This interpretation is further supported by the presence of caries-associated bacteria in the oral microbiome of Chagyrskaya Neanderthals, as evidenced by carious lesions found on the deciduous first upper molar designated Chagyrskaya 18 (Fig 18). Chagyrskaya 18 is a naturally exfoliated upper left deciduous first molar from a Neanderthal female of 9–11 years [61]. The crown exhibits approximately 50% occlusal wear. Post-depositional cracks are distributed across the entire crown surface. Pointed tartar deposits are present at the mesio-vestibular and disto-vestibular corners of the crown, running along the line of greatest crown expansion. There are two carious lesions on the crown. The first is a hole 0.6 mm in diameter and 0.5 mm deep in the fissure separating the metacone and protocone. Enamel is absent at the bottom of the hole, and the pathological process has spread to the upper dentin layer (Fig 19). The second carious lesion is localized in the center of the distal contact facet and represents an area of demineralized and depigmented enamel. In this case, dental disease is in the initial stage; the enamel layer is damaged, but the destruction has not yet reached the dentin surface.

The position of the drilled concavity and the toothpick groove on the Chagyrskaya 64 molar correspond precisely to these two types of pathological lesion locations. Two cases of caries in one small population is an unusual circumstance for Neanderthals, where carious lesions are very rare. To date, it has been observed in less than ten cases from the Palomas [62], Bau de l’Aubesier [63], De Nadale [64], and Kebara [65] sites. All these cases involved minor lesions of the enamel and dentin, not reaching the pulp chamber. Cases of deep crown lesions with pulp destruction have not yet been detected.

Key factors in the development of dental caries are the presence of cariogenic bacteria and easily fermentable carbohydrates in the oral cavity. The low incidence of this disease in Neanderthals has previously been explained either by a low carbohydrate diet or by the composition of their oral microflora, which may have lacked bacteria that cause caries in modern humans [66]. Isotopic analysis of tooth enamel from Chagyrskaya Neanderthals revealed that they had similar adaptive strategies to European Neanderthals, resulting in a similar trophic dietary ecology [2]. Accordingly, the development of dental caries may have been caused not so much by dietary factors as by the presence of specific cariogenic bacteria in the microbiome.

Evidence of the orientations and directionality of manipulation of lithic tools demonstrates advanced fine motor skills in the individual responsible for the modification of Chagyrskaya 64. This suggests that the presumed adaptation of Neanderthal hand structure for hafted tool manipulation – where primary force is applied through the wrist [67] – did not entail limitations in digital dexterity. This conclusion is further supported by analysis of bone retouchers from the Chagyrskaya Cave artifact assemblage, which revealed that Neanderthals predominantly used retouchers while holding them between two fingers, a technique requiring significant precision in movement [68].

Chagyrskaya 64 currently represents the earliest known evidence of intentional dental intervention. Previously, this distinction belonged to a case documented on the RM_3_ of a late Upper Paleolithic (Epigravettian) *H. sapiens* individual from Ripari Villabruna in northeastern Italy (14,160−13,820 cal yr BP). This tooth displayed traces of enamel caries scraping. Researchers suggest that the technique employed likely involved an adaptation of the well-documented toothpicking practice for levering and scratching purposes rather than drilling procedures [12,54].

The dental modifications observed on the Chagyrskaya 64 molar appear more advanced for two key reasons: first, they involved a more sophisticated drilling/rotating technique, which demands more complex and precise movements of the hand and fingers than does simple scraping, described by Oxilia et al. [12], and second, the interventions under certain conditions could be more effective. The Ripari Villabruna individual exhibits human-generated intervention marks primarily limited to the enamel, with minimal dentin involvement. Tomographic analysis of this tooth reveals significant retention of demineralized tissue [12], which would have facilitated continued disease progression and exacerbated pain responses. Scraping of the enamel would not have had a noticeable ameliorating effect on the intensity of felt pain.

The Chagyrskaya 64 molar exhibits complete pulp exposure. On the one hand, this could have caused pain for some time; on the other hand, pulp exposure would have accelerated nerve destruction, after which the pain should have disappeared. In the latter case, the removal of a large amount of coronal dentine would suggest potentially greater therapeutic efficacy. However, at present, we cannot say for certain whether the removal of a large volume of tissue was a conscious choice or resulted from the imperfection of the instrument used and the softness of the affected dentin.

Chagyrskaya 64 exhibits traces of two distinct types of interventions requiring different tools: 1 – drilling of the carious concavity and 2 – toothpick-like grooving. The use of drilling/rotating techniques – which demand complex finger manipulations – implies that Neanderthals possessed sophisticated cognitive capabilities. These would have included intuitive identification of pain sources, understanding of potential pain mitigation strategies, and deliberate selection of optimal dental intervention methods.

Recent discoveries – including Neanderthals’ production of modified bone tools, symbolic art, and evidence of patrilocal social organization [61,69–71] – have increasingly demonstrated cognitive convergence between Neanderthals and anatomically modern humans. Building on this, the Chagyrskaya 64 molar offers new insights into Neanderthal self-regulation, revealing their capacity for deliberate, sustained actions that required enduring pain – a behavior not documented in other higher primates, which typically rely on instinctive responses [5]. While genetic evidence confirms that Neanderthals exhibited heightened pain sensitivity compared to *Homo sapiens* [72], their ability to engage in goal-directed behaviors involving pain tolerance suggests advanced volitional control. It can be argued, therefore, that Neanderthals exhibited volitional strategic goal-setting behaviors that may have aligned more closely with those of *Homo sapiens* than with non-human primates.

## Conclusion

This study establishes compelling evidence for the earliest documented instance of invasive dental caries intervention in human evolutionary history, performed by Neanderthals approximately 59,000 years ago. Macroscopic, microscopic, and microtomographic analyses of the Chagyrskaya 64 lower left second molar, supplemented by experimental verification, confirm the anthropogenic origin of a substantial occlusal concavity resulting from deliberate ante-mortem manipulation.

The morphological and traceological features are indicative of a drilling/rotating executed with a lithic perforator, aimed at debriding carious tissue and accessing the pulp chamber. This finding represents a deliberate therapeutic intervention beyond palliative care. Concurrent evidence of pronounced toothpick grooving on the same element suggests a multifaceted approach to oral pathology management, employing distinct tools and methodologies. The integration of these techniques implies strategic selection and intentionality, transcending instinctive or purely palliative behaviors.

The technical proficiency required for this procedure – including precise digital manipulation, controlled force application, and tolerance of procedural discomfort – demonstrates advanced cognitive and sensorimotor capabilities. It reflects a capacity for causal reasoning, anticipatory planning, and volitional endurance, contradicting earlier assumptions regarding Neanderthal behavioral limitations. Furthermore, the operational success, evidenced by near-complete removal of decayed tissue and subsequent occlusal wear within the concavity, underscores its functional efficacy.

In summary, the Chagyrskaya 64 molar provides unprecedented insight into Middle Paleolithic healthcare paradigms. It attests to the presence of conceptually informed, invasive therapeutic practices among Neanderthals, significantly predating comparable evidence in *Homo sapiens*. This discovery not only enriches our understanding of Neanderthal behavioral complexity but also situates the roots of deliberate medical intervention deeper within the hominin lineage, challenging traditional distinctions in cognitive archaeology between anatomically archaic and modern members of *Homo sapiens* and highlighting their shared legacy of biological and cultural adaptations.

## Supporting information

S1 Fig Experiment 3, conducted on a modern *H. sapiens* molar. (JPG)

S2 Fig Experiment 1. a – general view of the tooth before experimental modification, b – general view of the occlusal surface with a concavity after the first stage of treatment, c – general view of the occlusal surface with a concavity and partially chipped crown after the second stage of treatment, d, e – macro-photos of linear striations on the walls of the concavity in the enamel layer, f, g – micro-photos of linear striations on the walls of the concavity, h – CT image of the experimental standard in five projections.
(TIF)

S3 Fig Experiment 2. a – general view of the molar occlusal surface before experimental modification, b – general view of the molar occlusal surface with concavities formed by manual rotation after the second stage of treatment, c, d, e – macro-photos of concavities and concavity walls in the enamel and dentin layer, f – CT image of the experimental tooth in six projections; c, d – fragment of enamel and dentin that began to chip off during the process of concavity deepening; e – third stage negative of dentin removal.
(TIF)

S4 Fig Experiment 3. a – general view of the molar occlusal surface before experimental modification, b – general view of the molar occlusal surface with depressions made by manual rotation after the second treatment stage, c – macro-photo of the three depressions (borders indicated as dotted lines), d – macro-photograph of the concavities with depression after the second stage of the experiment (the dotted line indicates the boundaries of the thin dentin layer fracture directly above the pulp cavity), e, f – macro-photographs of the two parts of the molar formed as a result of the fracture, g – CT image of the experimental tooth in six projections.
(TIF)

S5 Fig Macro- and micro- signs of rotating on enamel and dentin. a – macro-photo of the wall of the concavity of experimental tooth #3, b – macro-photo of processing traces on the walls of the concavity (light upper part – enamel, darker part below – dentin), c – micro-photo of linear striations on the surface of dentin in the lower part of the concavity. The traces are oriented differently because the area was processed by scraping, d – micro-photo of rotating traces on the surface of dentin.
(TIF)

S6 Fig Use-wear traces on experimental lithic (jasper) perforator. (TIF)

S7 Fig Diagnostic features of toothpick use documented on different *Homo* individuals. a – interproximal wear groove detected on a *Homo erectus* molar from Olduvai Gorge, Tanzania (OH 60). Source: [37]; b – interproximal groove recorded on the distal side of the left second lower molar (SD-1327i) of a *Homo neandertalensis* from El Sidrón Cave, Asturias, Spain. Source: [38]; c – interproximal groove identified on the mesial facet of a right second lower molar of a *Homo neandertalensis* from Rochelot Cave (Saint-Amant-de-Bonnieure, Charente, France). Source: [39]; d – groove with multiple microstriations recorded on molariform tooth fragments (left side) of *Homo erectus* found in Olduvai Gorge, Tanzania (OH 62). Source: [14].
(JPG)

S1 Text Supporting information text and references. (DOCX)
