# Correction: Earliest evidence for invasive mitigation of dental caries by Neanderthals

**DOI:** 10.1371/journal.pone.0351227

**Published:** 2026-06-05

**Authors:** Alisa V. Zubova, Lydia V. Zotkina, John W. Olsen, Alexander M. Kulkov, Vyacheslav G. Moiseyev, Anna A. Malyutina, Roman V. Davydov, Sergey V. Markin, Eugene A. Maksimovskiy, Pavel V. Chistyakov, Andrey I. Krivoshapkin, Ksenia A. Kolobova

There is an error in affiliation 7 for authors Andrey I. Krivoshapkin and Ksenia A. Kolobova. The correct affiliation 7 is: Archeology and Paleoecology of the Stone Age in Central Asia (APSACA) Lab, Institute of Anthropology, Uzbekistan Academy of Sciences, Tashkent, Uzbekistan.

## References

[1] Zubova AV, Zotkina LV, Olsen JW, Kulkov AM, Moiseyev VG, Malyutina AA, et al. Earliest evidence for invasive mitigation of dental caries by Neanderthals. PLoS One. 2026;21(5):e0347662. doi: 10.1371/journal.pone.0347662 42127021 PMC13170851

