# Supplementary figures and images for: Earliest evidence for invasive mitigation of dental caries by Neanderthals
Source: PLoS One. 2026 May 13;21(5):e0347662. doi: 10.1371/journal.pone.0347662 (PMC13170851; doi:10.1371/journal.pone.0347662)

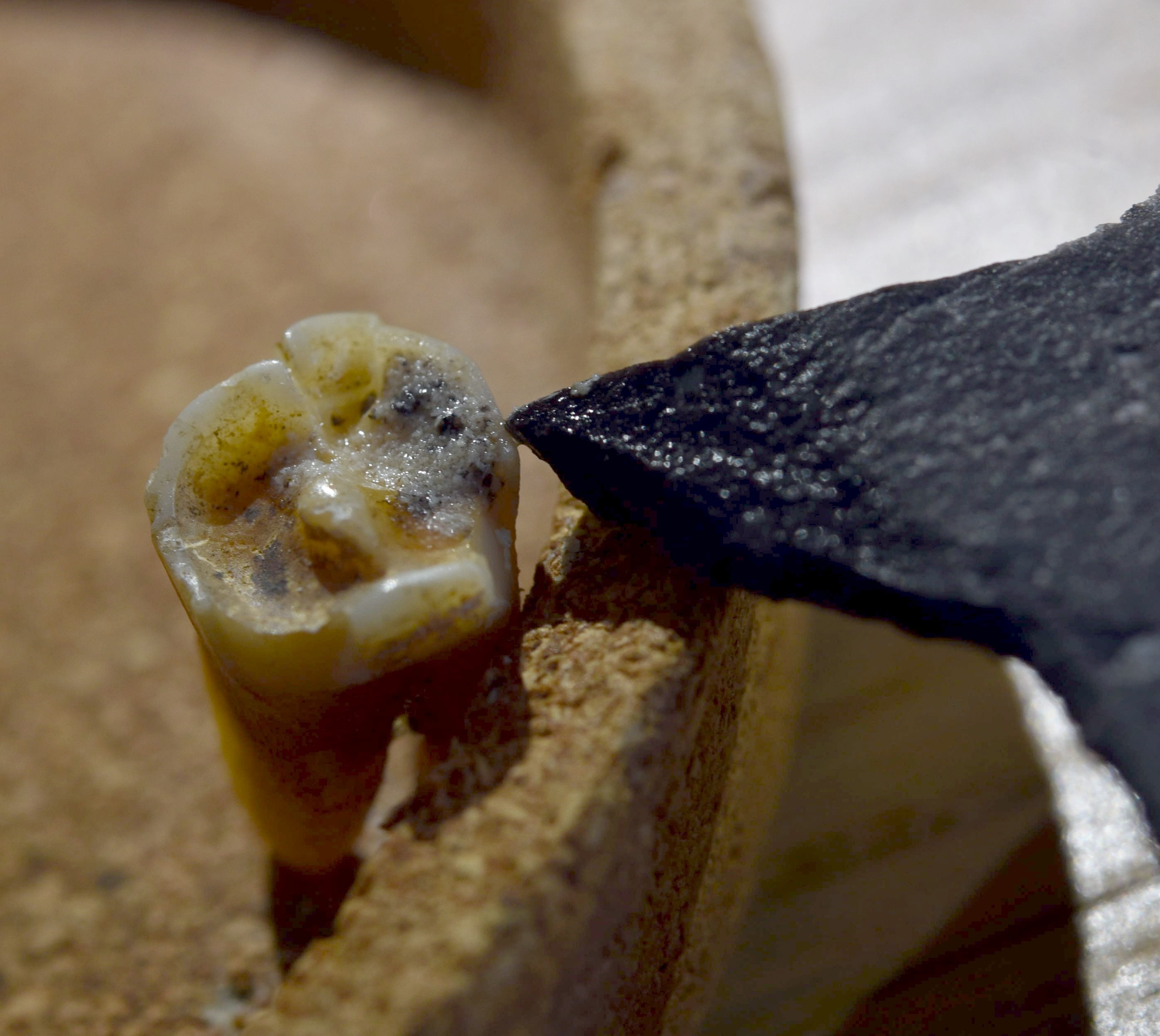

Supplement: S1 Fig — (JPG) [file pone.0347662.s001.jpg]

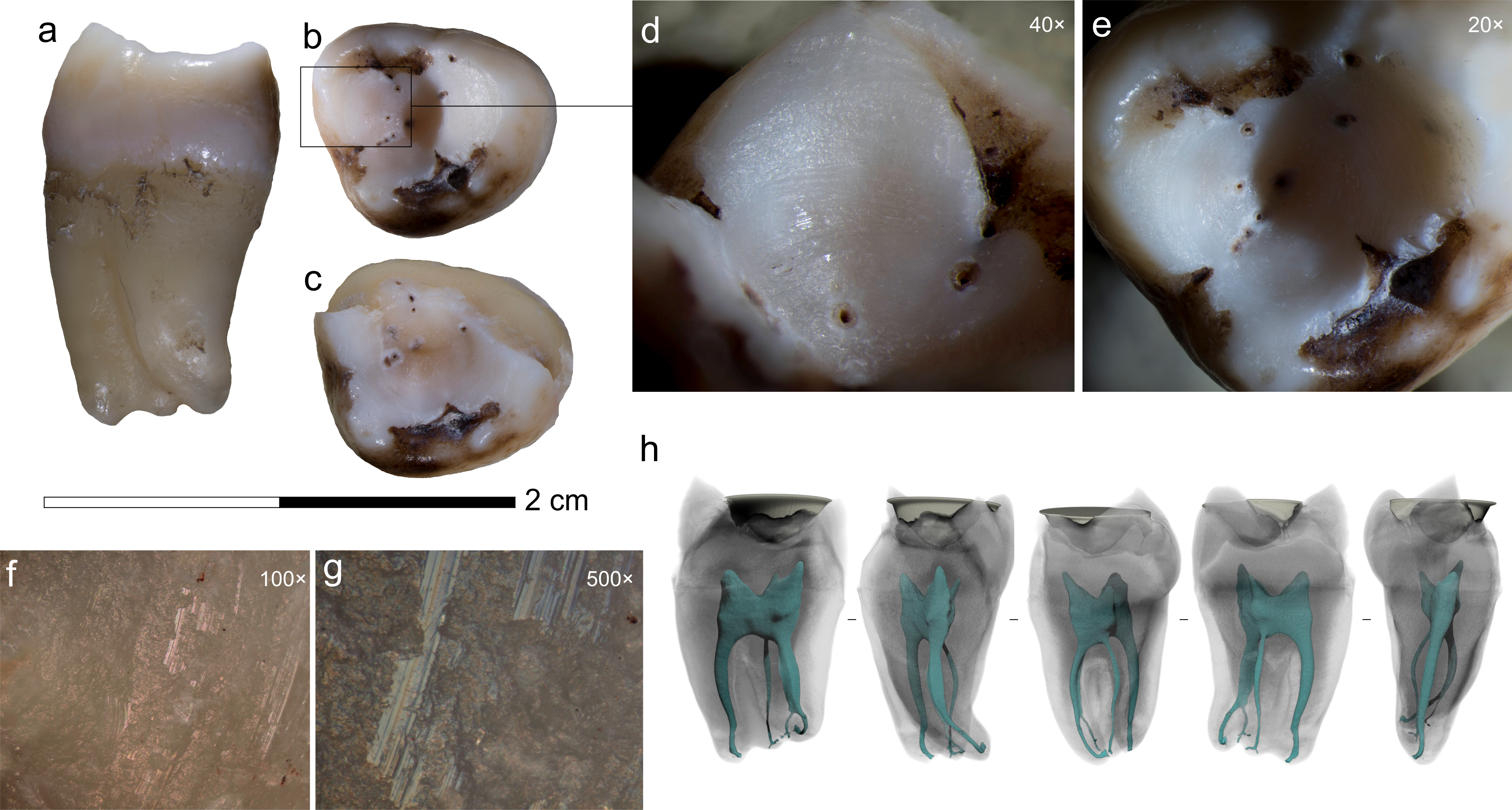

Supplement: S2 Fig — a – general view of the tooth before experimental modification, b – general view of the occlusal surface with a concavity after the first stage of treatment, c – general view of the occlusal surface with a concavity and partially chipped crown after the second stage of treatment, d, e – macro-photos of linear striations on the walls of the concavity in the enamel layer, f, g – micro-photos of linear striations on the walls of the concavity, h – CT image of the experimental standard in five projections. (TIF) [file pone.0347662.s002.tif]

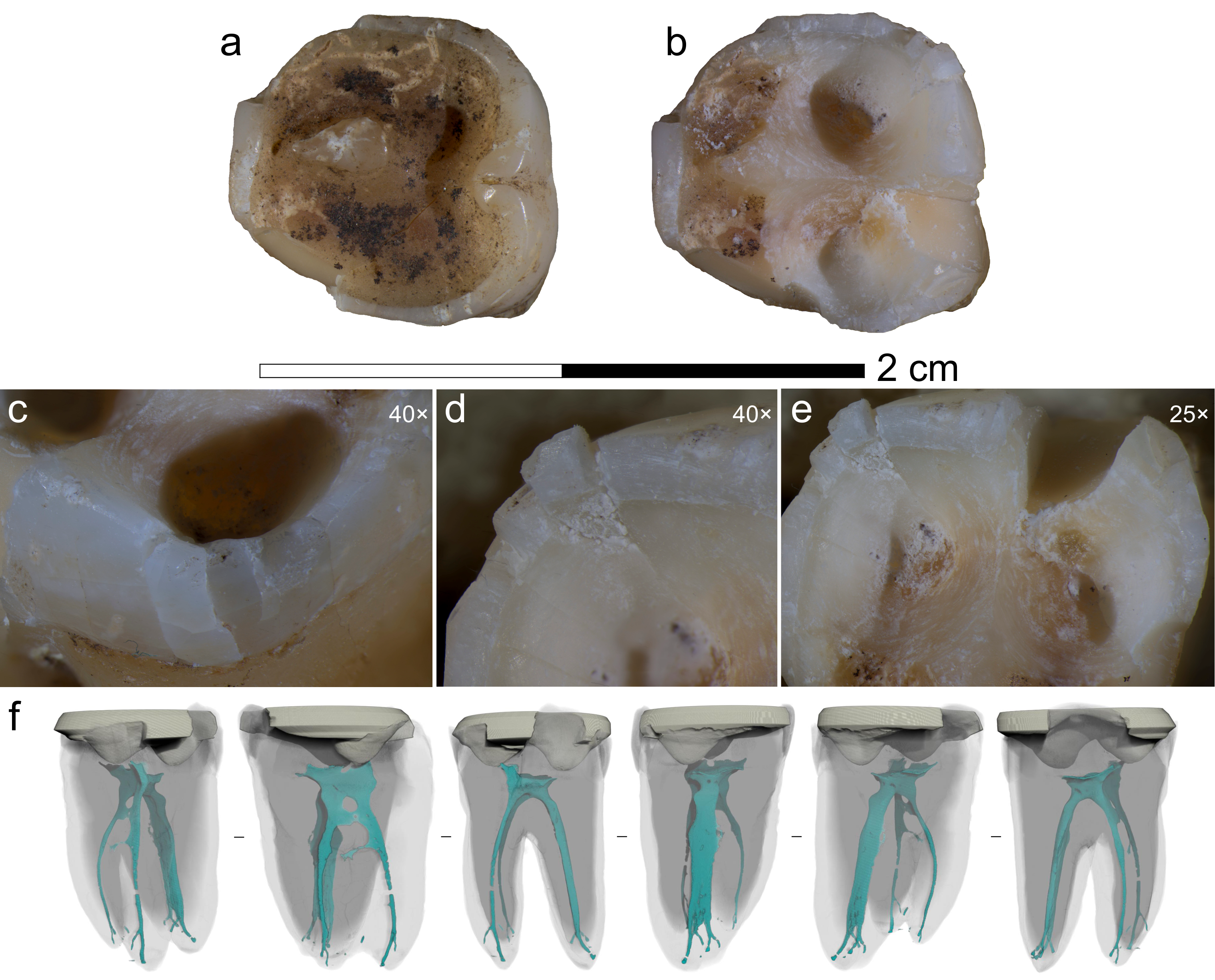

Supplement: S3 Fig — a – general view of the molar occlusal surface before experimental modification, b – general view of the molar occlusal surface with concavities formed by manual rotation after the second stage of treatment, c, d, e – macro-photos of concavities and concavity walls in the enamel and dentin layer, f – CT image of the experimental tooth in six projections; c, d – fragment of enamel and dentin that began to chip off during the process of concavity deepening; e – third stage negative of dentin removal. (TIF) [file pone.0347662.s003.tif]

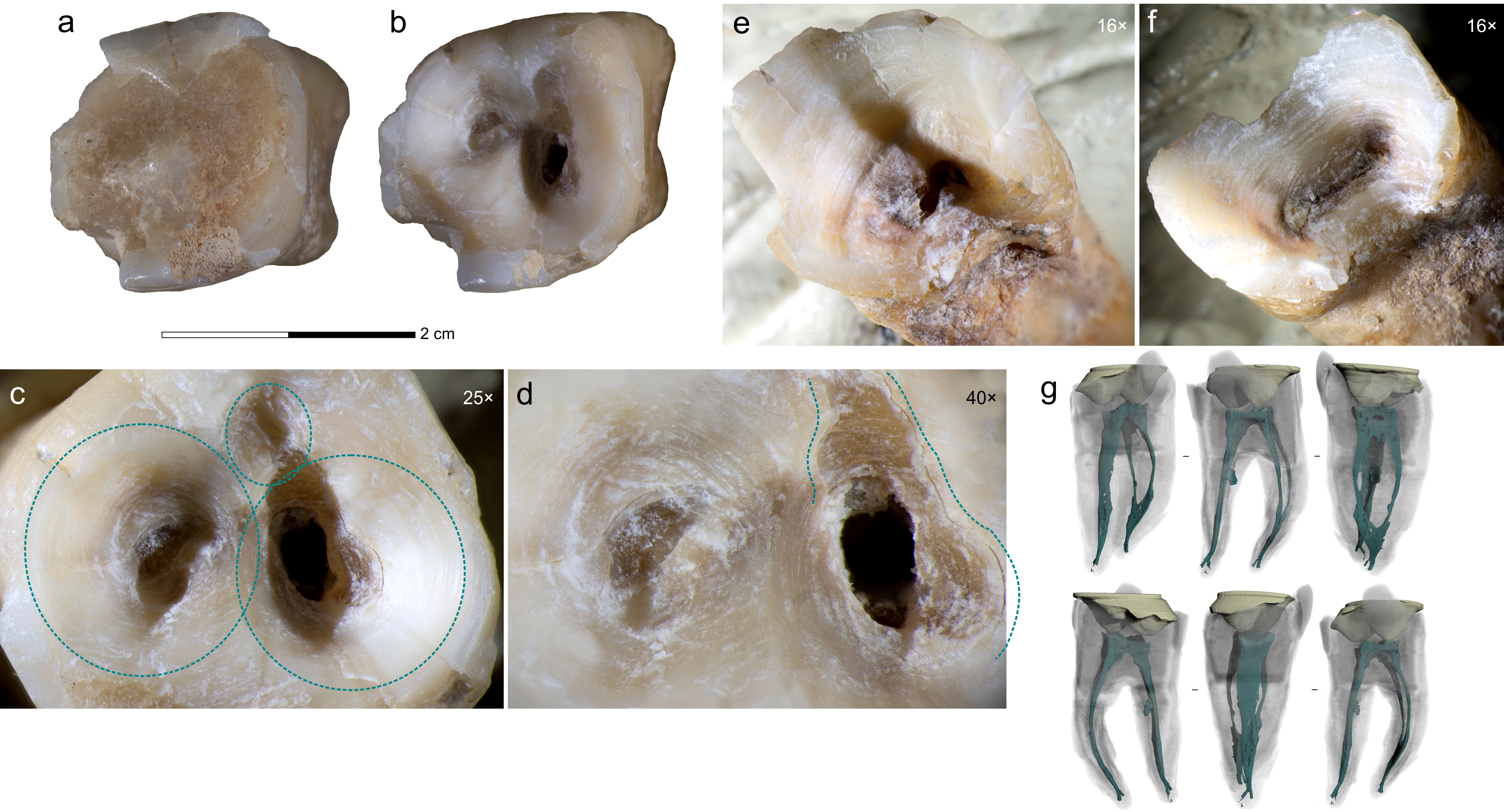

Supplement: S4 Fig — a – general view of the molar occlusal surface before experimental modification, b – general view of the molar occlusal surface with depressions made by manual rotation after the second treatment stage, c – macro-photo of the three depressions (borders indicated as dotted lines), d – macro-photograph of the concavities with depression after the second stage of the experiment (the dotted line indicates the boundaries of the thin dentin layer fracture directly above the pulp cavity), e, f – macro-photographs of the two parts of the molar formed as a result of the fracture, g – CT image of the experimental tooth in six projections. (TIF) [file pone.0347662.s004.tif]

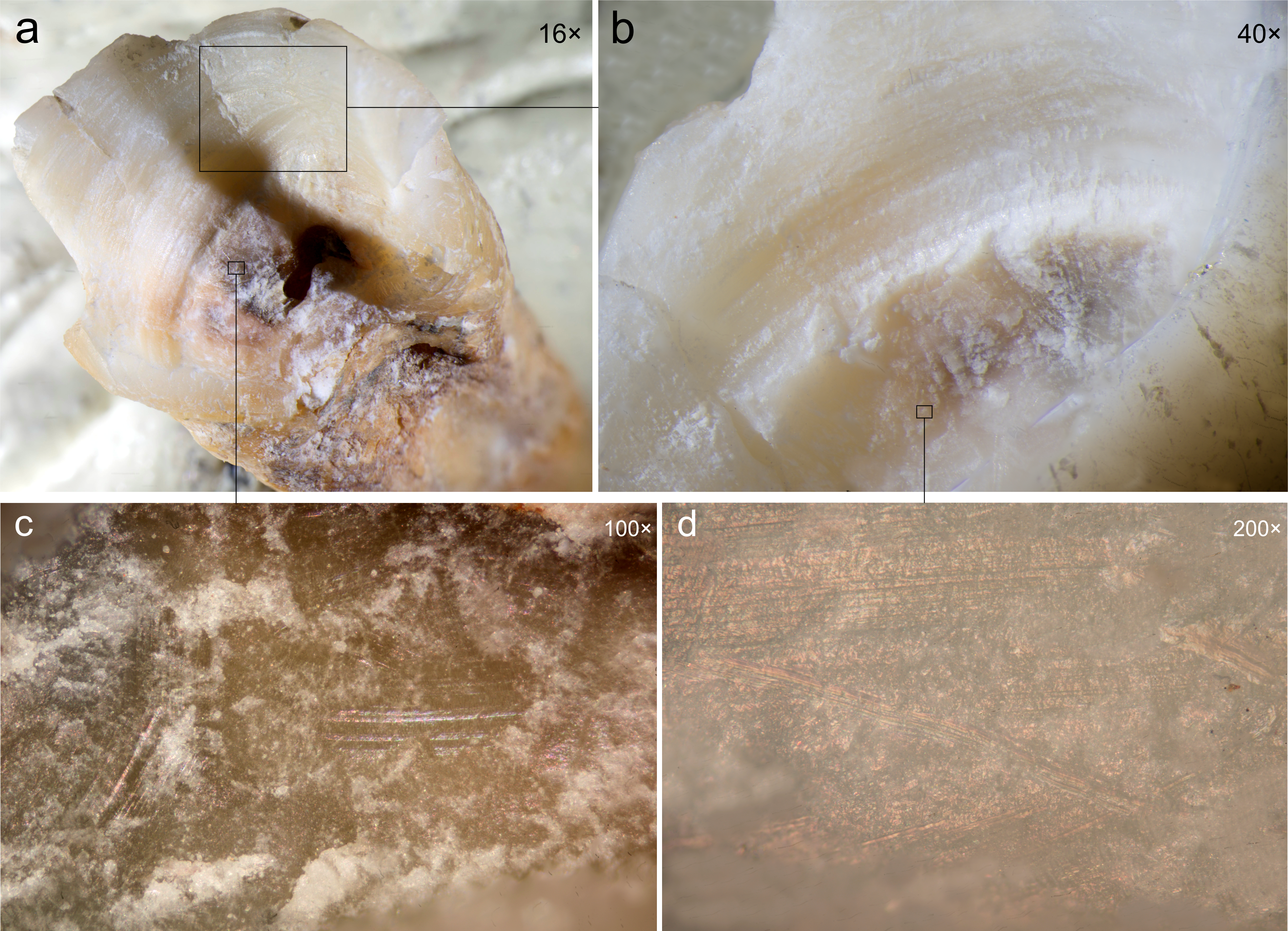

Supplement: S5 Fig — a – macro-photo of the wall of the concavity of experimental tooth #3, b – macro-photo of processing traces on the walls of the concavity (light upper part – enamel, darker part below – dentin), c – micro-photo of linear striations on the surface of dentin in the lower part of the concavity. The traces are oriented differently because the area was processed by scraping, d – micro-photo of rotating traces on the surface of dentin. (TIF) [file pone.0347662.s005.tif]

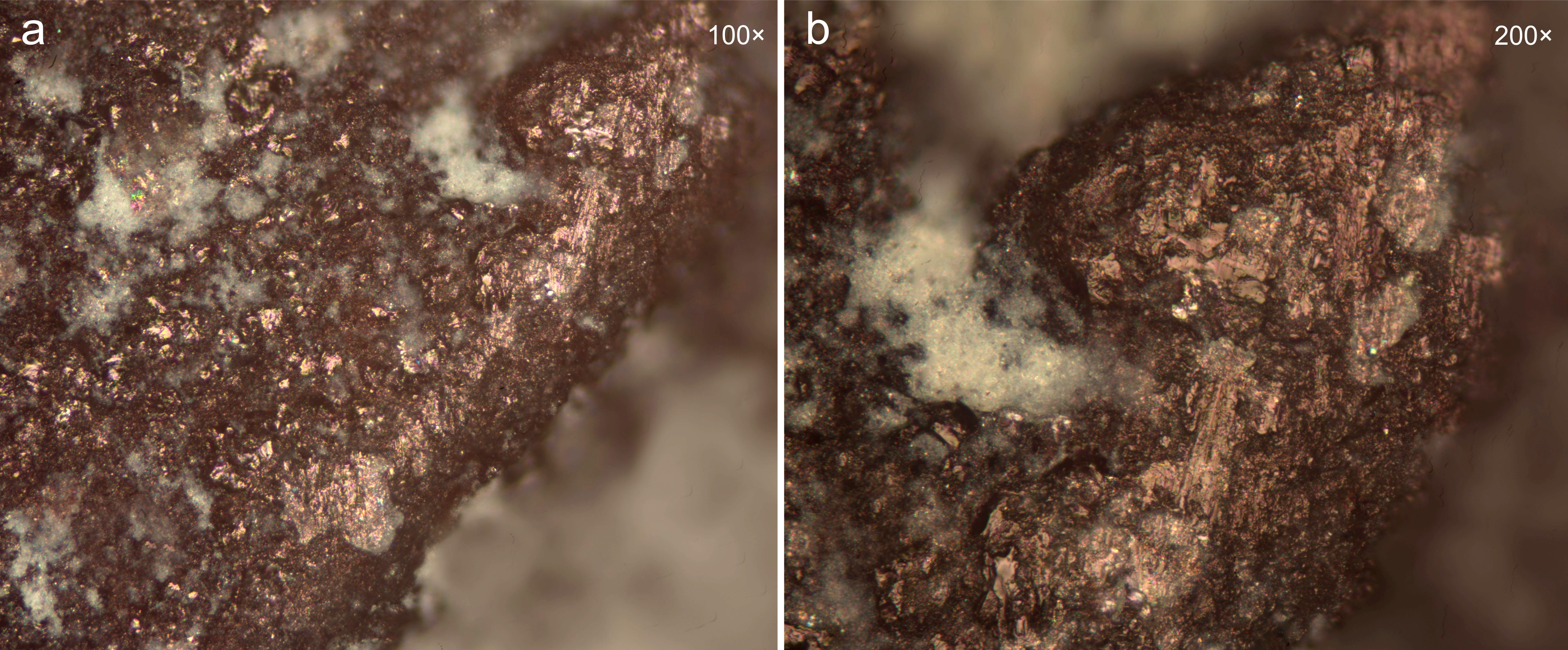

Supplement: S6 Fig — (TIF) [file pone.0347662.s006.tif]

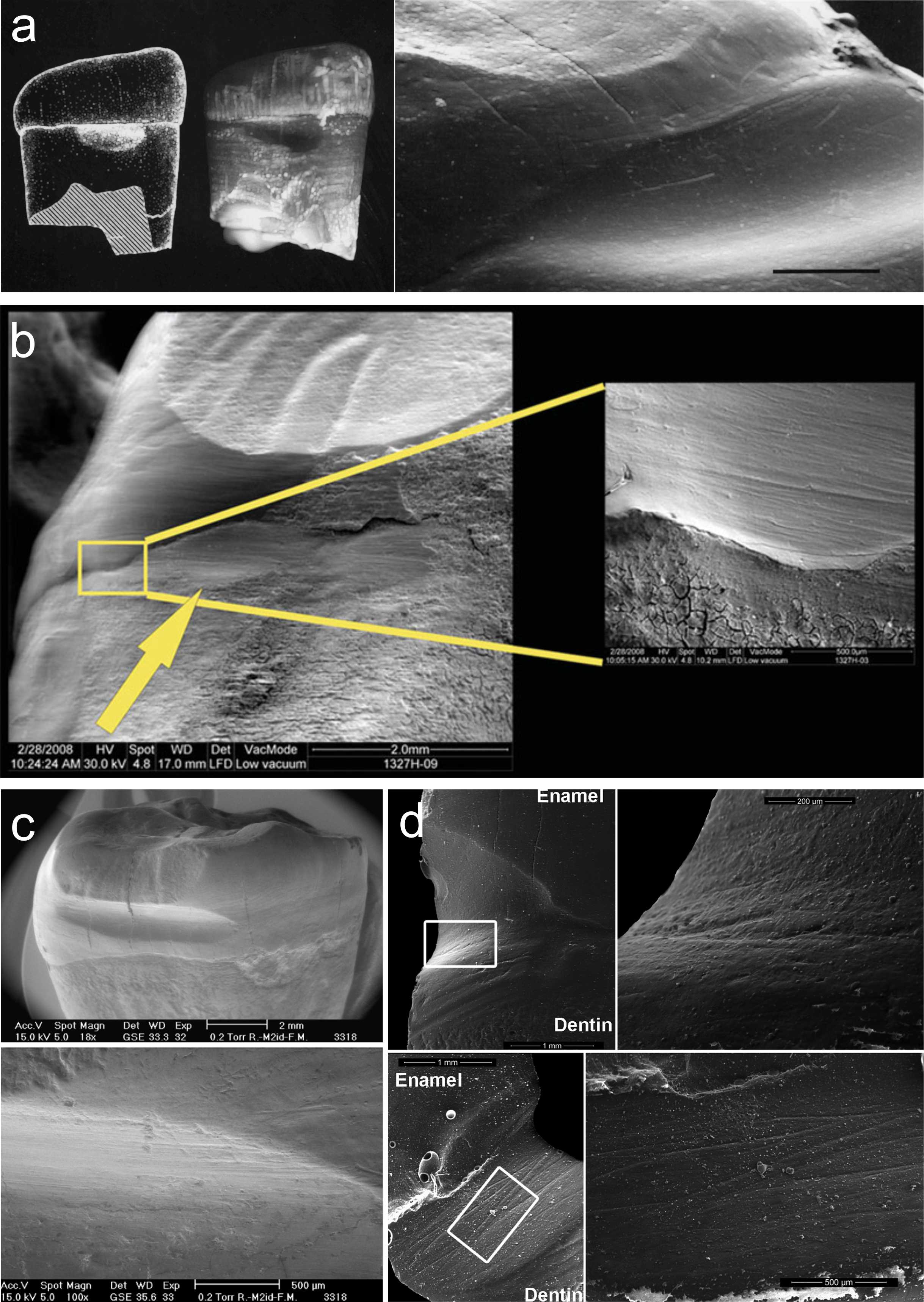

Supplement: S7 Fig — a – interproximal wear groove detected on a Homo erectus molar from Olduvai Gorge, Tanzania (OH 60). Source: [37]; b – interproximal groove recorded on the distal side of the left second lower molar (SD-1327i) of a Homo neandertalensis from El Sidrón Cave, Asturias, Spain. Source: [38]; c – interproximal groove identified on the mesial facet of a right second lower molar of a Homo neandertalensis from Rochelot Cave (Saint-Amant-de-Bonnieure, Charente, France). Source: [39]; d – groove with multiple microstriations recorded on molariform tooth fragments (left side) of Homo erectus found in Olduvai Gorge, Tanzania (OH 62). Source: [14]. (JPG) [file pone.0347662.s007.jpg]
