# Supplementary material for: Earliest evidence for invasive mitigation of dental caries by Neanderthals
Source: PLoS One. 2026 May 13;21(5):e0347662. doi: 10.1371/journal.pone.0347662 (PMC13170851; doi:10.1371/journal.pone.0347662)
Supplement: S1 Text — (DOCX) [file pone.0347662.s008.docx]

Supporting Information Text

**Description of the experimental teeth, conditions, course of experiments and observations obtained**

Supporting Information **References**

1. Ungar PS, Grine FE, Teaford MF, Pérez-Pérez A. A review of interproximal wear grooves on fossil hominin teeth with new evidence from Olduvai Gorge. Arch Oral Biol. 2001; 46(3): 285–292.
2. Radini A, Buckley S, Rosas A, Estalrrich A, de la Rasilla M, Hardy K. Neanderthals, trees and dental calculus: new evidence from El Sidrón. Antiquity. 2016; 90(350): 290–301. doi:10.15184/aqy.2016.21
3. d'Incau E, Couture C, Maureille B. Human tooth wear in the past and the present: Tribological mechanisms, scoring systems, dental and skeletal compensations. Arch Oral Biol. 2012; 57(3): 214–229. doi:10.1016/j.archoralbio.2011.08.021
4. Estalrrich A, Alarcón JA, Rosas A. Toothpicking in early *Homo* OH 62 from Olduvai Gorge (Tanzania): An indirect evidence of intensive meat consumption? J Hum Evol. 2020; 143: 102769. doi:10.1016/j.jhevol.2020.102769
